# Differential tissue deformability underlies fluid pressure driven shape divergence of the avian embryonic brain and spinal cord

**DOI:** 10.1016/j.devcel.2025.04.010

**Published:** 2025-05-09

**Authors:** Susannah B.P. McLaren, Shi-Lei Xue, Siyuan Ding, Alexander K. Winkel, Oscar Baldwin, Shreya Dwarakacherla, Kristian Franze, Edouard Hannezo, Fengzhu Xiong

**Affiliations:** 1 Wellcome Trust / CRUK Gurdon Institute, University of Cambridge, Cambridge, UK; 2 Department of Physiology, Development and Neuroscience, University of Cambridge, Cambridge, UK; 3 Institute of Science and Technology Austria, Klosterneuburg, Austria; 4 Department of Materials Science and Engineering, School of Engineering, Westlake University, Hangzhou, China; 5 Friedrich-Alexander-Universität Erlangen-Nürnberg, Erlangen, Germany; 6 Max-Planck-Zentrum für Physik und Medizin, Erlangen, Germany

## Abstract

An enlarged brain underlies the complex central nervous system of vertebrates. The dramatic expansion of the brain that diverges its shape from the spinal cord follows neural tube closure during embryonic development. Here, we show that this differential deformation is encoded by a pre-pattern of tissue material properties in chicken embryos. Using magnetic droplets and atomic force microscopy, we demonstrate that the dorsal hindbrain is more fluid than the dorsal spinal cord, resulting in a thinning versus a resisting response to increasing lumen pressure, respectively. The dorsal hindbrain exhibits reduced apical actin and a disorganised laminin matrix consistent with tissue fluidisation. Blocking the activity of neural-crest-associated matrix metalloproteinases inhibits hindbrain expansion. Transplanting dorsal hindbrain cells to the spinal cord can locally create an expanded brain-like morphology in some cases. Our findings raise questions in vertebrate head evolution and suggest a general role of mechanical pre-patterning in sculpting epithelial tubes.

## Introduction

Morphogenesis, the process through which organisms, organs and tissues achieve their shape, plays a crucial role in determining biological function. This is exemplified in the vertebrate central nervous system, with an expanded brain enabling the complex behaviours of vertebrate animals that promote their adaptation in diverse ecological niches^1–3^. Initial morphological differences between the brain and spinal cord emerge as the antero-posteriorly patterned neural plate folds to form the neural tube^4–7^ during embryonic development. Neural plate folding also brings together the presumptive neural crest, located in the elevating neural folds^7^, which subsequently undergo an epithelial to mesenchymal transition at the dorsal surface of the closing neural tube, migrating away laterally and ventrally to other locations where they contribute to a wide range of tissues^8,9,10^. Following neural tube closure, a dramatic expansion of the brain lumen diverges its shape from the spinal cord^11^, setting their distinct morphologies for further development^12,13^. How the brain and the spinal cord expand differentially remains unclear. In the avian brain, the expansion is known to depend on the hydrostatic pressure from the neural tube lumen^14,15^. For example, a positive lumen pressure is required for the global circumferential expansion of the brain neuroepithelium as the midbrain and forebrain bend away from the posteriorly connected spinal cord^15,16^. This pressure arises as the sealed neural tube starts to make the cerebrospinal fluid by active transport of Na+ and secretion of proteins and proteoglycans into the lumen, establishing an osmotic gradient drawing water inside^17^. By linking the pressure to stress-associated growth, a model was proposed to explain the expansion of the brain chambers while the contrictions between them that express aligned F-actin deepen^15,16^. However, the material property of the neural epithelium, the key ingredient for understanding tissue shape responses to external stresses like the lumen pressure, remains unknown.

Here, using the chicken embryo as a model, we show that the hindbrain expands through dorsal tissue thinning under a positive hydrostatic pressure from the neural tube lumen while the dorsal spinal cord resists this pressure and maintains its shape. Using magnetic droplets and atomic force microscopy, we reveal that the dorsal tissue in the hindbrain is more fluid than in the spinal cord. The dorsal hindbrain harbours more migratory neural crest cells^18^ and exhibits reduced apical actin and a disorganised laminin matrix compared to the dorsal spinal cord. Blocking the activity of matrix metalloproteinases inhibited dorsal tissue thinning while not affecting tissue growth through cell proliferation, leading to abnormal brain morphology. Transplanting early dorsal hindbrain cells to the spinal cord created a region with expanded brain-like morphology including a thinned-out roof in a small number of cases, suggesting that these cells may be sufficient to drive local dorsal tissue thinning. These findings reveal that a mechanically pre-patterned neural tube subjected to extrinsic force diverges the brain and spinal cord in shape. This mechanism may be employed generally in other epithelial morphogenesis processes. By connecting the behaviour of the cranial neural crest cells, a key population implicated in head formation, with one of the earliest sources of long-range force in embryo development, neural tube lumen pressure, our findings imply a co-origin of cellular mehcanisms that underlie an enlarged brain and an elaborated head during vertebrate evolution.

## Results and Discussion

### Hindbrain expansion is driven by neural tube lumen pressure

**Figure 1:**
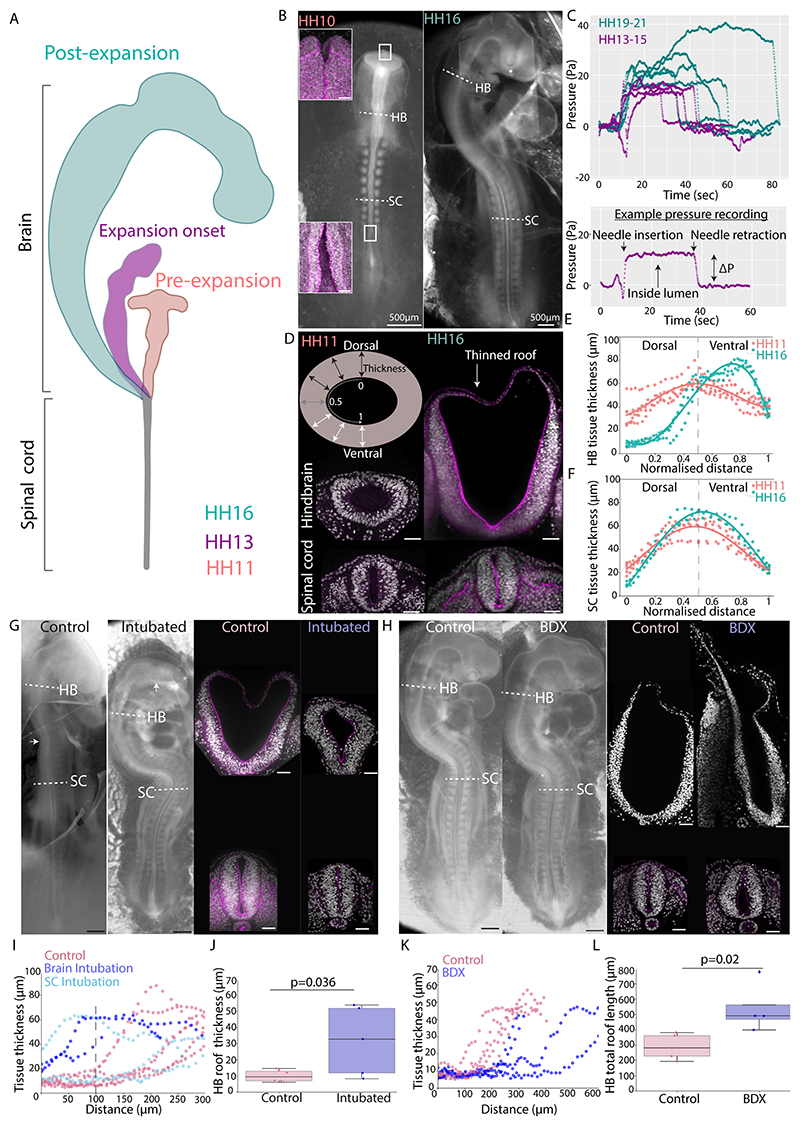
Neural tube lumen pressure drives hindbrain and spinal cord shape divergence (A) Schematic depicting brain expansion during chicken embryo development with Hamburger and Hamilton stages. (B) Bright-field images of chicken embryos prior to (HH10) and following brain expansion (HH16) and confocal images of the early anterior and posterior neuropore at the level of the white boxes. Actin is shown in magenta throughout. Dashed lines indicate level of cross-sectioned regions with the otic vesicle as a landmark. (C) Neural tube lumen pressure readings in embryos at the onset of (n=4) and following brain expansion (n=5). (D) Confocal images of neural tube cross-sections and schematic showing tissue thickness measurement approach and normalisation to the dorsal and ventral midpoint. (E and F) Apical-basal tissue thickness along the hindbrain and spinal cord lumen circumference (n=6 HH11 embryos, n=3 HH16 embryos). (G) Bright-field images of control and intubated embryos at ~20 hours post-intubation and confocal images of corresponding hindbrain and spinal cord cross-sections. (H) Bright-field images of control and β-D-xyloside (BDX) treated embryos ~20h post treatment and corresponding confocal images of hindbrain and spinal cord cross-sections. Some distortion occurs due to fixation and sectioning. (I) Tissue thickness along the hindbrain circumference from dorsal to ventral in control (n=6) and intubated (n=5) embryos. (J) Hindbrain roof thickness in control (n=6) and intubated (n=5) embryos (p=0.036, t-test). (K) Tissue thickness along the hindbrain circumference in control (n=5) and BDX treated (n=4) embryos. (L) Hindbrain total roof length in control (n=5) and BDX treated (n=4) embryos(p=0.02, t-test). Black scale bars are 500μm, white scale bars are 50μm unless otherwise stated. See also Figure S1.

In avian embryos, the volumetric expansion of the brain over a relatively stable spinal cord takes place shortly after neural tube closure (Figure 1A). Prior to brain expansion (before Hamburg-Hamilton (HH) stage 13^11^), the neural tube has large openings at both the anterior and posterior ends that allow fluid to move between the tube lumen and its surroundings (Figure 1B), implying a lack of pressure differential. These openings then narrow and close concomitant with the onset of brain expansion around HH13^19^. Consistent with pioneering studies^20,21^, which reported positive brain lumen pressures (albeit variable in absolute magnitude, probably depending on the experimental instruments and conditions), we recorded a lumen pressure of ~15Pa in the initial stages of brain expansion (HH13-15). Spinal cord lumen pressure was not directly measurable with our probes due to the small lumen size, so we instead tested the continuity of the lumen to assess potential pressure differentials along the neural tube. We guided injected ferrofluid droplets from the brain into the spinal cord using a magnetic field (Figure S1A-B). At the transition between the hindbrain and spinal cord, the droplet thins significantly, in agreement with the previously observed narrowing of the spinal cord lumen in these stages and indicating a possible occulusion^22^. To test this, we injected dyes in both ends of the neural tube. The dye was found in the opposite far ends of the brain and spinal cord from the injection sites (Figure S1C). Using our pressure controller^23^ to impose a high pressure in the brain, we found an immediate lumen expansion in the spinal cord (Figure S1D). These results suggest that the neural tube lumen is a continuous fluid filled cavity and unlikely maintains large pressure differentials between the brain and the spinal cord. We measured an increase in lumen pressure to ~25Pa as expansion progressed (HH19-21), during stages at which the brain and spinal cord lumen are known to be well connected^24^ (Figure 1C and S1E). To assess how tissue morphology changes following this pressure increase, we measured neural tube thickness around the circumference of the lumen in cross sections of the spinal cord and the hindbrain prior to and after brain expansion (HH11 and HH16, respectively. Figure 1D-F). The hindbrain lies directly anterior to the spinal cord and has a simple tubular morphology making this region a good system to investigate mechanisms responsible for shape divergence between the brain and the spinal cord. To ensure consistency and control for the antero-posterior variation of the hindbrain shape, the hindbrain region flanked by the landmark otic vesicles was used for hindbrain sections. We observed that dorsal hindbrain tissue approximately halved in thickness and formed a single-cell thick epithelium concomitant with brain expansion, whilst little dorsal thinning or tissue shape change occured in the spinal cord (Figure S1F and G).

To test the role of lumen pressure in hindbrain dorsal thinning, we intubated^16^ embryos in the brain or spinal cord just prior to brain expansion (HH11), leaving the tube lumen connected to the embryo’s surroundings to equate the pressure (Figure S1H). Although we observed an increase in hindbrain cross-sectional area, in line with proliferation and tissue growth, dorsal hindbrain thinning and expansion were inhibited in intubated embryos following 20 hours of incubation (Figure 1G, I and J). Not only brain-intubated but also a spinal cord-intubated embryo showed inhibited hindbrain thinning, further suggesting that the lumen is connected along the anterior-posterior axis of the neural tube and the tissues experience similar hydrostatic pressure. The intubation phenotype cannot be explained by tissue damage as tubes that penetrate the lumen to exit from both sides do not affect hindbrain thinning and expansion (Figure S1H). We attempted the converse experiment by connecting our intubation needle to a pressure column, which allows for dynamic pressure modulation in the neural tube lumen^23^. However, we could not achieve stable long-term pressure increase, likely due to the high pressure differential causing leakage at the puncture site. As an alternative method, we treated embryos with β-D-xyloside (BDX)^25^, which osmotically increases lumen pressure by ~30%^15^. This led to an increase in expansion and extension of the thinned dorsal roof in the hindbrain and a moderately enlarged spinal cord lumen with no dorsal thinning (Figure 1H, K and L). Together these findings demonstrate that neural tube lumen pressure drives dorsal tissue thinning in the hindbrain but is resisted in the dorsal spinal cord, leading to brain expansion relative to the spinal cord.

### The hindbrain and spinal cord have different material properties

**Figure 2:**
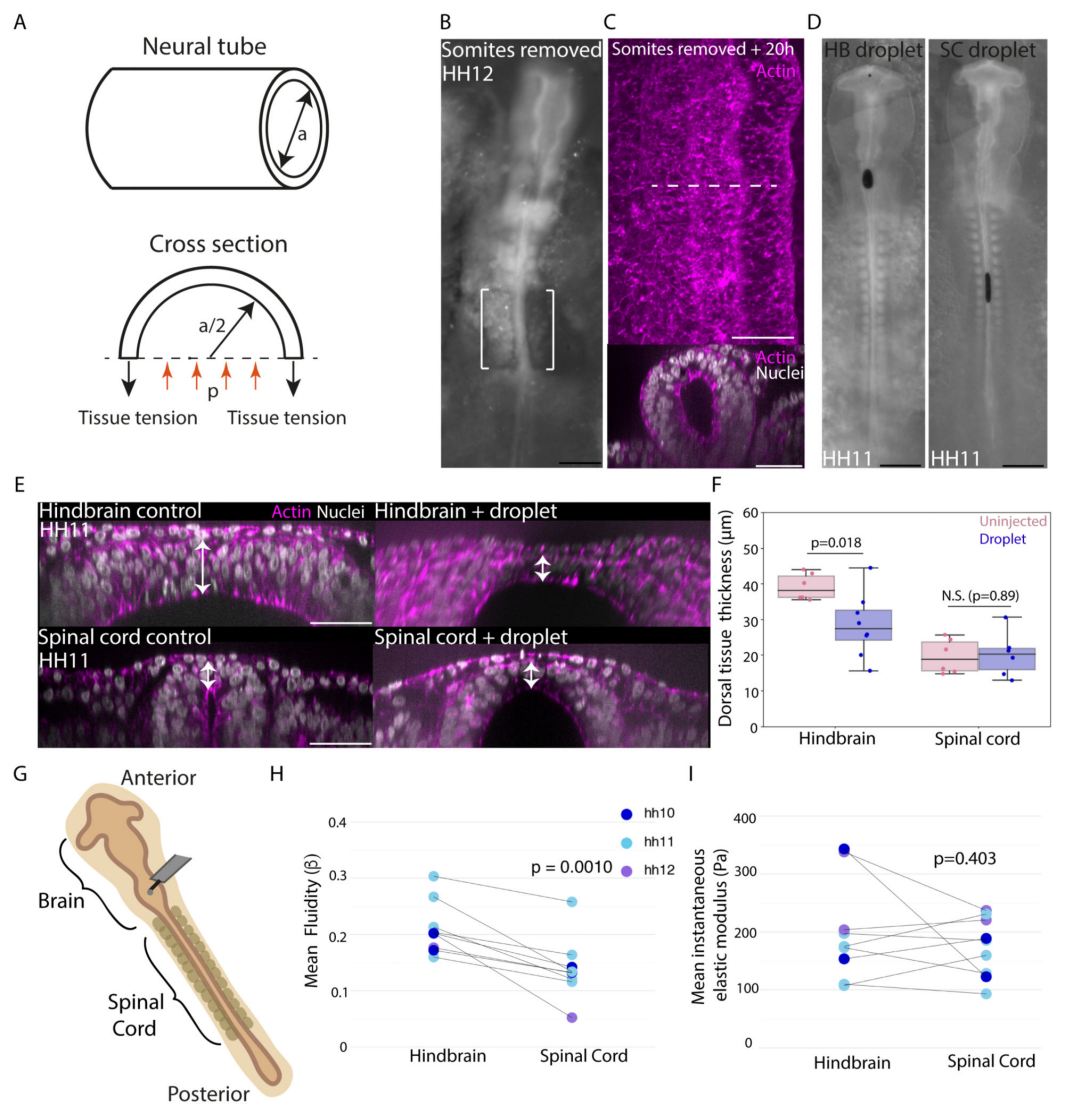
The dorsal hindbrain is more deformable than the dorsal spinal cord prior to brain expansion (A) Schematic illustrating forces exerted on a tubular tissue. (B) Bright-field image of a pre-brain expansion embryo with posterior somites and anterior presomitic mesoderm removed. (C) Confocal images of the dorsal surface and cross-section of the spinal cord ~20h post somite-removal. (D) Widefield images of HH11 stage embryos with ferrofluid droplet injections in the hindbrain or spinal cord lumen. (E) Confocal images showing cross-sectional views of HH10-11 stage hindbrain and spinal cord regions without and with injected ferrofluid droplets. Arrows indicate thickness measurements. (F) Quantification of hindbrain and spinal cord roof thickness in uninjected (n=6 hindbrain, n=6 spinal cord) and droplet injected (n=8 hindbrain, n=6 spinal cord) embryos (p=0.018 and 0.89, t-tests). (G) Schematic depicting atomic force microscopy measurement set up. (H and I) Mean Beta value (corresponding to fluidity) and mean instantaneous elastic modulus of the hindbrain and spinal cord in HH10-12 embryos (n=9) (p=0.001 and 0.403, t-tests). Black scale bars are 500μm. White scale bars are 50μm. See also Figure S2 and Data S1.

The differential thinning of dorsal tissue between the hindbrain and the spinal cord may result from a few scenarios. First, different initial tissue curvature at the onset of pressure increase (Figure 1D) can lead to diverging dynamics of thinning and volume expansion. Generically, forces exerted on a tubular tissue are proportional to both the luminal fluid pressure and the tissue radius. Driven by a similar pressure, the tubular region with a greater initial radius (lower curvature) would then display larger tissue forces and expansion (Figure 2A), which could be consistent with the shape dynamics observed in the hindbrain and spinal cord. To experimentally test this possibility, we modified spinal cord lumen shape prior to brain expansion by surgically removing somites and anterior presomitic mesoderm on either side of the spinal cord (Figure 2B). This operation releases tissue confinement from the somites (Figure S2A) and the dorsal non-neural ectoderm as it was cut through during somite ablation. After ~20 hours of incubation, these embryos healed and exhibited reduced spinal cord dorsal curvature and in one case clear rounding of the spinal cord cross section (Figure 2C, S2B-C). However, this did not lead to the formation of a thinned-out roof, as was observed in the hindbrain (Figure S2D). These results suggest that the higher curvature of the spinal cord dorsal region is not sufficient to explain the lack of dorsal tissue thinning in the spinal cord. At the same time, this experiment ruled out a second alternative model in which local confinement from neighbouring tissues (such as somites and the presomitic mesoderm) might modulate neural tube shape (^26^ and Figure S2A) by constraining lumen expansion of the spinal cord relative to the hindbrain. This led us to explore a third hypothesis: that the differential expansion of the brain and spinal cord was due to the dorsal tissue of the hindbrain being more deformable than that of the spinal cord, enabling local thinning under hydrostatic pressure. To test whether the material properties of the hindbrain and spinal cord dorsal tissue differed before the onset of increased lumen pressure, we injected ferrofluid oil droplets into the forebrain of HH11 embryos and positioned the droplets in either the hindbrain or spinal cord using a magnetic field (Figure 2D). We then observed the resulting tissue deformation from the pressure generated by the droplets as they try to round up under surface tension after removal of the magnetic field (Figure 2E). The droplets showed a higher curvature at the droplet-lumen interface in the spinal cord, indicating a higher pressure was exerted on the spinal cord tissue than the hindbrain by this mechanical perturbation^27^ (Figure S2E). Still, the thickness change of dorsal tissue at the droplet site was greater in the hindbrain than in the spinal cord (Figure 2F), indicating that hindbrain dorsal tissue was more deformable. Furthermore, observing droplet shape change over time revealed a slow rounding process occurring over hours (Movies S1 and S2), orders of magnitude longer than would be expected from droplet surface tension and viscosity alone, which provided an opportunity to estimate the effective long-term viscous response of the surrounding tissue (Figure S2F). To do this, we took a modelling approach, which has been successfully exploited for ferrofluid droplets in other studies^28^ but not in this tubular configuration. We modelled the neural tube as a viscoelastic Maxwell medium with differing geometry between the brain and spinal cord (Data S1, supplementary theory note). By fitting droplet shape dynamics to the model, we found that the estimated hindbrain viscosity was much lower than the spinal cord and that a lumen pressure of 10’s of Pa is capable of driving hindbrain expansion over long timescales, consistent with our pressure measurements (Figure 1C). As an independent validation of our findings on tissue mechanical properties, we used an atomic force microscopy (AFM) cantilever to indent the dorsal region of the hindbrain and spinal cord (Figure 2G) in the same embryos and measured tissue relaxation in response to a sustained force (50 or 75nN). Using a power-law model^29^, we found that the dorsal hindbrain had a higher fluidity than that of the spinal cord prior to brain expansion on short timescales, whilst no significant difference in instantaneous elastic modulus was detected (Figure 2H and I). While *in vivo* AFM measurements may contain a contribution of differences from other non-local structures such as the extracellular matrix (ECM) anchoring the neural tube, these results show a more deformable hindbrain tissue environment. Although a quantitative comparison of the AFM creep and the ferrofluid droplet measurements is difficult because of their distinct time scales, qualitatively both methods uncovered the same tendency of hindbrain tissue to become more fluid than spinal cord tissue. Furthermore, incorporating a less viscous dorsal region into our neural tube model generated tissue thinning and expansion dynamics consistent with experimental observations of hindbrain morphogenesis (Data S1, section 4). Together, these findings support the hypothesis that the dorsal hindbrain deforms more than the dorsal spinal cord under lumen pressure through viscous thinning over time. Importantly, the mechanical differences between the tissues are readily present by HH11, prior to neural tube closure and pressure onset.

### Neural crest mediated matrix remodelling as a mechanism for brain tissue fluidisation

**Figure 3:**
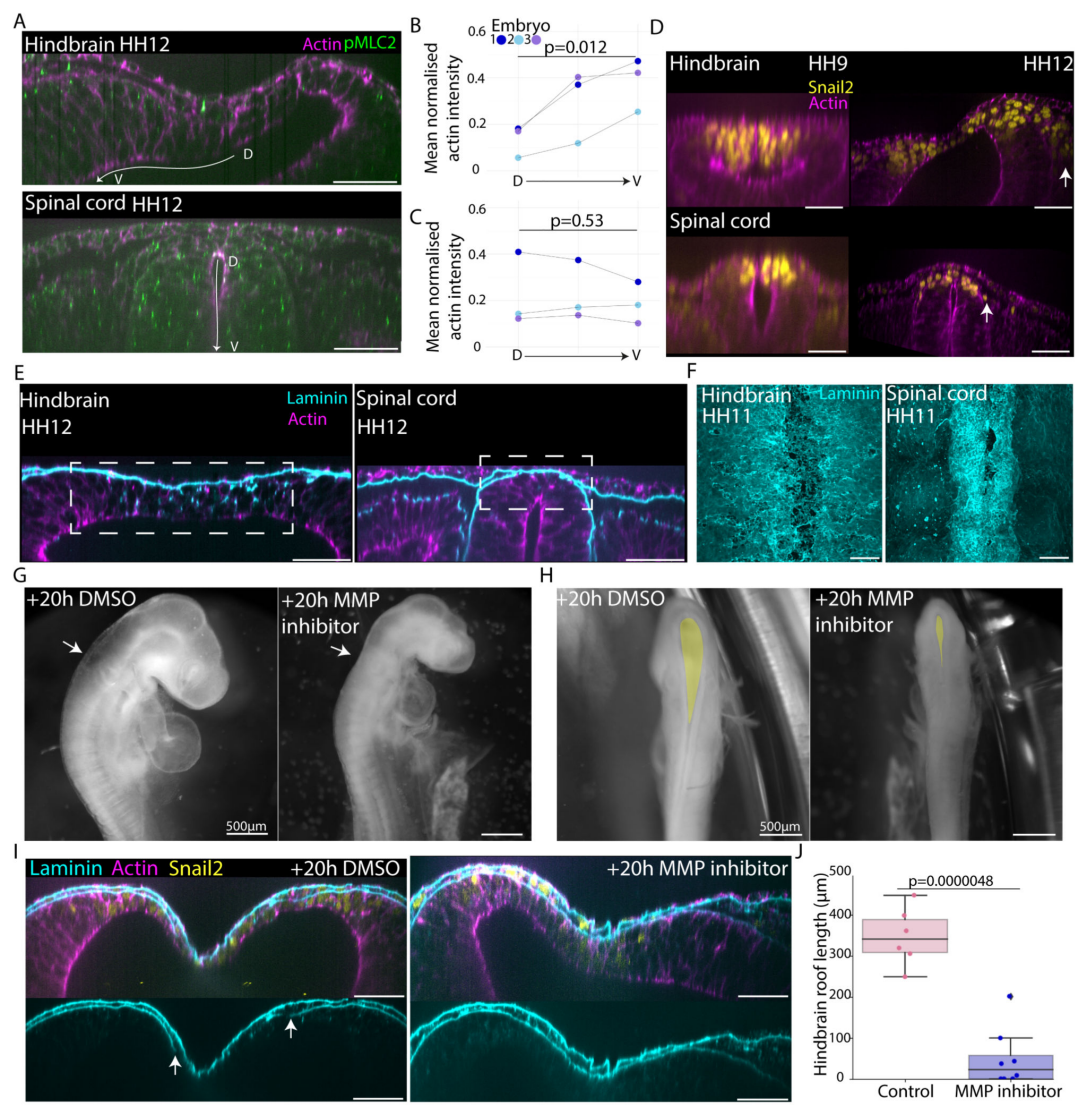
Differential neural crest behaviour underlies the acquisition of a thinned out and expanded dorsal hindbrain roof (A) Confocal images of actin and phosphorylated myosin light chain kinase II in the hindbrain and spinal cord of HH12 stage embryos. (B and C) Quantification of apical actin signal intensity along the lumen circumference in the hindbrain and spinal cord of HH11-HH12 stage embryos (n=3) (p=0.012 and 0.53, t-tests). (D) Confocal images showing Snail2+ cell migration in the hindbrain and spinal cord cross sections of embryos progressing towards brain-expansion stages (HH9 to HH12). (E) Confocal images showing laminin and actin organisation in cross section views of the hindbrain and spinal cord of HH12 stage embryos. (F) Confocal images showing laminin organisation at the dorsal surface in a 3D rendering of the hindbrain and spinal cord of HH11 stage embryos. (G) Bright-field images of embryos treated with Dimethyl sulfoxide (DMSO) or MMP inhibitor at HH11 and incubated for 20 hours. Arrows indicate hindbrain at level of otic vesicle. (H) Dorsal views of DMSO and MMP inhibitor treated embryos. Yellow highlight fills the brain dorsal surface. (I) Confocal images showing the dorsal hindbrain of control and MMP inhibitor treated embryos, ~20 hours post treatment. Bottom panels show laminin surface with arrows indicating breaks in laminin continuity. (J) Hindbrain roof length in control (n=6) and inhibitor treated (n=8) embryos (p=0.0000048, t-test). White scale bars are 50μm unless otherwise stated. See also Figure S3.

To investigate the mechanism of dorsal tissue deformability, we explored the cellular organisation of the tissue at stages leading up to brain expansion (HH9-12). The dorsal neural tube tissue is occupied by neural crest cells that reorganise their actomyosin cytoskeleton as they undergo an epithelial-to-mesenchymal transition (EMT) and delaminate from the tube dorsal surface as the walls of the neural plate fuse dorsally^30,31,32^. The dynamics of neural crest delamination between cranial and trunk regions is known to differ^33,34^, making them an exciting candidate for regulating tissue intrinsic differences between the dorsal hindbrain and spinal cord. We observed clear apical organisation of actin and myosin within the dorsal spinal cord in HH12 embryos, however this organisation was largely absent in the neural crest domain of the hindbrain (Figure 3A-C, S3A). This change in cytoskeleton organisation indicated a transition away from an epithelial cell state to a more mesenchymal cell state in the dorsal hindbrain. A key player in this process is the zinc-finger transcription factor Snail2 which is expressed in the pre-migratory and early migrating neural crest^35,36^. Using Snail2 as a marker, we observed that the dynamics of neural crest delamination and migration at the dorsal surface of the neural tube differed between the hindbrain and the spinal cord leading up to brain expansion, with Snail2+ cells migrating in a cell-by-cell fashion in spinal cord regions, whilst those in the hindbrain migrate in a collective wave (Figure 3D and S3B), consistent with previous studies^33,37^. Neural crest cells utilise matrix metalloproteases (MMPs) to remodel the surrounding ECM^38,39^. ECM architecture plays an important role in determining tissue material properties, with higher levels of ECM organisation conferring greater stiffness (or elasticity) and more disordered matrix organisation leading to softening (or a more viscous behaviour)^40,41^, which contributes to long-term deformability.

This led us to investigate ECM organisation in the hindbrain and the spinal cord as a potential mediator of tissue deformability. Cross-sectional views showed that laminin was redistributed throughout the dorsal hindbrain in the premigratory neural crest domain, whilst comparatively little redistribution was observed in the dorsal spinal cord (Figure 3E and S3C). This was associated with a more sparce dorsal laminin coating of the hindbrain compared to the spinal cord (Figure 3F and S3D and E). Notably, the basal laminin layer of the covering skin stays intact and continuous in both regions. These findings suggest that ECM is being actively reorganised and redistributed out of the plane of the basement membrane to a greater extent in the hindbrain.

**Figure 4:**
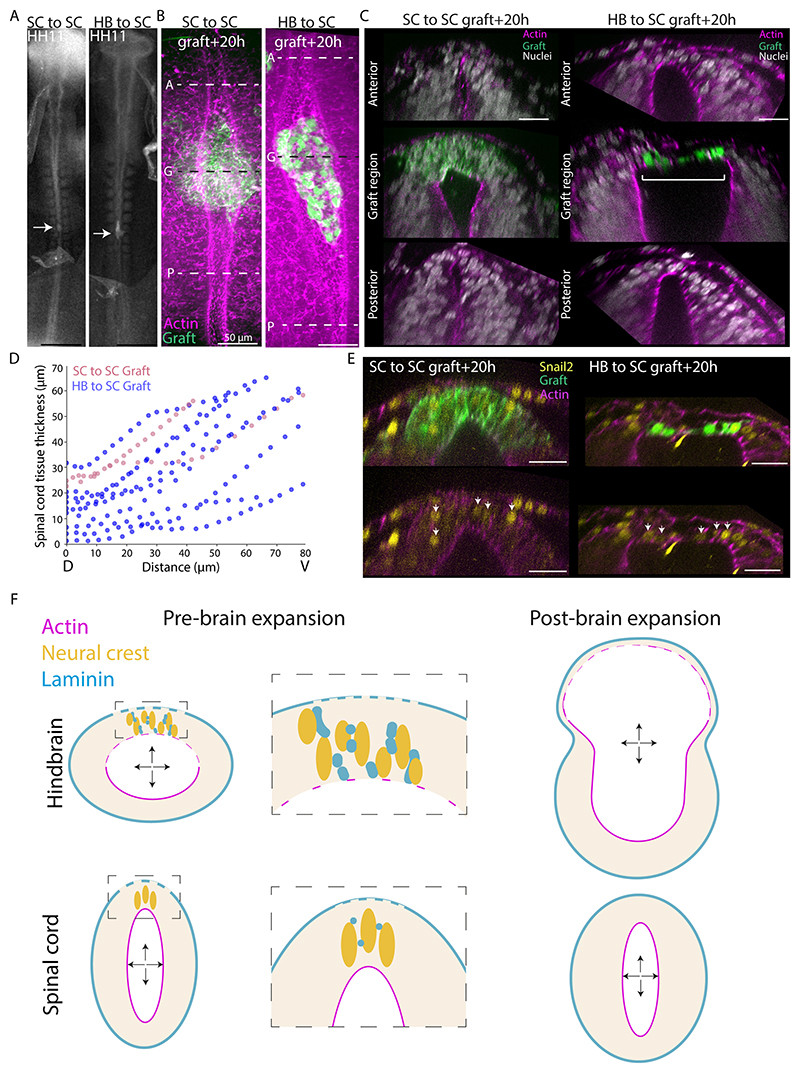
Hindbrain premigratory neural crest cells may be sufficient to generate neural tube expansion under lumen pressure (A) Bright-field images of pre-brain expansion stage embryos with spinal cord-to-spinal cord graft and a hindbrain-to-spinal cord graft. (B and C) Confocal images showing dorsal surface of graft integration ~20 hours post-grafting and cross-sectional views at the levels corresponding to dashed lines in (B). White bracket highlights the thinned-out roof in the graft region. (D) Spinal cord tissue thickness in the region with spinal cord (n=2) or hindbrain (n=9) grafted cells. (E) Confocal images showing Snail2+ cells in graft regions. Arrows indicate Snail2+ cells within the GFP+ graft. (F) Model of brain expansion relative to the spinal cord. A greater extent of premigratory neural crest cell mesenchymal behaviour and corresponding extracellular matrix remodelling underlies a more deformable dorsal roof in the early hindbrain compared to the spinal cord. This allows the hindbrain roof to deform more under internal lumen pressure, driving hindbrain expansion relative to the spinal cord during early embryo development. Black scale bars are 500μm. White scale bars are 25μm unless otherwise stated. See also Figure S4.

To test whether ECM remodelling plays a role in dorsal hindbrain thinning, we inhibited MMP activity in embryos prior to brain expansion stages using an MMP-specific small molecule inhibitor (GM6001, 1000μM). Brain expansion was reduced in inhibitor treated embryos and a smaller surface area of the dorsal hindbrain was apparent (Figure 3G-H). In embryos with obvious observable developmental defects (0/6 control, 4/8 inhibitor treated), the hindbrains did not undergo dorsal thinning and in some cases aggregated a mass of Snail2+ cells within the lumen (Figure S3F). In embryos that developed normally overall (6/6 control, 4/8 inhibitor treated), the hindbrains of the treated embryos showed thicker or shorter dorsal roofs than those of the controls (Figure 3I, J and S3F). The laminin surface covering the basal extent of the dorsal hindbrain appeared less disrupted in embryos with inhibited roof thinning (Figure 3I and S3G and H) and the dorsal tissue was still populated with Snail2+ cells, suggesting that neural crest specification continued in the presence of the inhibitor. Together, these data are consistent with the idea that neural crest mesenchymal behaviour and subsequent ECM remodelling is required for thinning and expansion of the dorsal hindbrain. To test the intrinsic ability of early hindbrain dorsal cells to generate a thinned roof under lumen pressure, we performed a grafting experiment, moving a small piece of dorsal hindbrain (or spinal cord as a control) tissue from a GFP+ embryo to the pre-cut dorsal region of the spinal cord of a wildtype host before the onset of lumen pressure (Figure 4A). Re-integration of the graft to reform a closed neural tube that would continue normal morphogenesis was expected to be rare given the difficulty of positioning and maintaining the grafts contact in such a small incision site. After multiple rounds of grafting followed by overnight culture, we accumulated 9 embryos with different degrees of hindbrain dorsal tissue integration into the spinal cord, and 2 spinal cord-to-spinal cord controls (Figure 4B, S4A and S4B). The control embryos exhibited a thick dorsal region at the grafted site (Figure 4C, D and S4B, 2/2 embryos). Interestingly, 3 hindbrain-to-spinal cord grafted embryos exhibited dorsal tissue thinning in the grafted region (Figure 4C, D and S4D). In the cleanest graft where the donor tissue completely replaced the host dorsal region, we found a distinctively thinned out dorsal roof and hindbrain-like morphology specific to the grafted region (Figure 4C), with a thicker spinal cord-like host dorsal region present on the anterior and posterior sides of the graft. Cells in the grafted region were Snail2+ (Figure 4E) and the thinned-out roof coincided with loss of apical actin localisation (Figure S4C), suggesting that the grafted region contained premigratory neural crest cells undergoing EMT. This striking ability of Snail2+ dorsal hindbrain cells to form a thinned-out and elongated roof in the spinal cord region suggests that hindbrain premigratory neural crest cells may be sufficient to drive dorsal tissue thinning in response to lumen pressure.

Taken together, our findings support a mechanism where deformability of the neural tube dorsal tissue and ECM organisation underlie the distinct shape changes of the brain and the spinal cord under a shared lumen pressure (Figure 4F). This mechanism can work indepdently or in concert with other proposed mechanisms such as active constriction and differential growth^16^. The mechanical differences likely result from different behaviours of the neural crest cells, which are known to exhibit different signalling, metabolism and fates between cranial and trunk levels^33,42^. Our study thus implies a closely coordinated initial developmental step coupling brain expansion and neural crest EMT, both essential contributors to the formation and evolution of the vertebrate head^43^. These findings are in agreement with classic chimera grafting experiments in which reduced dorsal roof thinning and elongation can be observed in hindbrain regions lacking neural crest cells^44^. While ECM remodelling likely facilitates neural crest EMT and migration, dorsal neural tissue fluidisation may also promote migration by enhancing the stiffness gradient that was shown to guide neural crest cells^45^. Future studies will be needed to pin down the exact developmental and spatial window of competence in which brain expansion can occur. Expanded early brain morphology is highly conserved between chicken and human embryos^46^, and irregularly shaped mesenchymal cells in the dorsal hindbrain have also been observed to contribute to the squamous roof plate in zebrafish embryos^47^, suggesting that neural crest mediated dorsal tissue fluidisation may contribute to brain expansion in other species. Future work will resolve the detailed molecular and cellular regulation of neural crest behaviour differences between the brain and spinal cord that underpin dorsal neural tissue mechanics and identify genetic and environmental factors that may cause developmental defects associated with aberrant brain expansion through this mechanism. More sensitive tools are required for spatial-temporal profiling of lumen pressure. Combined with identification of factors that drive pressure increase, which may include transient changes in spinal cord lumen shape^22^ and lumen fluid composition, these tools will reveal the mechanisms underlying pressure regulation. Given the role of lumen pressure in driving morphogenesis of a variety of epithelial tissues and organs^48,49,50,51^ our findings here in the neural tube imply a general strategy for creating diverse biological shapes via a mechanical property pre-pattern before the onset of changes in fluid pressure.

### Limitations of the Study

In intubated embryos that showed decreased dorsal thinning we observed an increase in tissue cross sectional area consistent with normal tissue growth. However, in addition to pressure, the lumen fluid’s chemical composition was likely also changed by connecting the lumen to the external environment. We have not investigated fluid composition change and cannot rule out its potential contribution to dorsal thinning.

Whether differences in neural crest cell behaviour between the trunk and brain region of the neural tube are intrinsic or depend on an external signal from their environment remains to be determined. We were unable to achieve enough successful graftings of early stage dorsal spinal cord cells into the early dorsal hindbrain in combination with overnight incubation to test this due to lack of tissue integration.

### Resource availability

#### Lead Contact

Further information and requests for resources and reagents should be directed to and will be fulfilled by the Lead Contact, Fengzhu Xiong (fx220@cam.ac.uk).

#### Materials Availability

This study did not generate new unique reagents.

## STAR Methods

### Experimental model and study participant details

#### Egg lines and embryo culture

Wild type fertilised chicken eggs were obtained from MedEgg Inc., GFP+ eggs (Cytoplasmic ^52^ and Membrane^53^) were obtained from the National Avian Research Facility (NARF) at University of Edinburgh. Eggs were kept in a fridge at 14°C for storage and in a 37.5°C humidified (40%-60%) incubator (Brinsea) during incubation. No animal protocol under the UK Animals (Scientific Procedures) Act 1986 was required for the chicken embryo stages investigated (incubated under 2 weeks, or 2/3 of the gestation time). *Ex ovo* culture was performed using a modified EC culture^54^ protocol as described^55^. For *in ovo* operations, egg shells were windowed with surgical scissors after removing some albumen. A small volume of Chinese calligraphy ink (YiDeGe, Black) was then injected underneath the embryo to help with visualisation. After performing surgical, mechanical or chemical perturbations, eggs were resealed with clear plastic tape and returned to incubation.

### Method details

#### Pressure measurement

Pressure measurements were performed as detailed in^23^. Briefly, a glass microneedle connected to a pressure sensor was inserted into the neural tube lumen of chicken embryos at varying stages of development. Pressure readings were continuously recorded to include the pressure just outside the lumen, after insertion into the lumen, and after retraction from the lumen for each experiment. The pressure difference between the outside and inside of the lumen was taken as the neural tube lumen pressure. Measurements where no stable pressure reading could be obtained inside the neural tube lumen, likely due to needle clogging by tissue debris during the insertion, were excluded from analysis.

#### Pressure modulation

Intubation experiments were performed by inserting an open-ended short glass capillary tube into the forebrain or spinal cord region of HH10-12 stage embryos. Control experiments were performed by either inserting a glass capillary into tissues just neighbouring the neural tube, inserting a very narrow glass capillary and not submerging it in fluid with the embryo, or inserting a long capillary that penetrates the neural tube and comes out from the other side. β-D-xyloside (BDX) experiments were performed by adding either DMSO or BDX to embryo culture medium at a final concentration of 2mM in L15 media. In all cases embryos were cultured overnight *ex ovo*. Only embryos that displayed continued development were included in the analysis. The effect of intubation was examined after 20 hours, beyond which the embryos were not followed due to high variability and low survival rate.

#### Assessment of lumen continuity

Fluorescently labelled Dextran was injected into the neural tube lumen of HH11-13 stage embryos using a Nanojet injector. The fluorescent signal was imaged following injection to assess how far the dye travelled along the neural tube lumen. The dye was observed to spread anteriorly into the brain region and posteriorly into the posterior spinal cord when injected into the spinal cord lumen mid-way along the trunk.

#### Removal of confining tissues

Posterior somites and anterior presomitic mesoderm were removed from embryos *in ovo* using a sharpened tungsten needle or pulled glass capillary. The vitelline membrane was peeled back and cuts were made at the interface between the spinal cord region of the neural tube and posterior somites and anterior presomitic mesoderm. Somites and presomitic mesoderm were peeled away to leave the posterior spinal cord unconfined on its sides. Eggs were then resealed and incubated overnight. Embryos that showed a good degree of healing and no obvious spinal cord ruptures were included in analysis.

#### Grafting experiments

Small patches of dorsal hindbrain or spinal cord cells from GFP positive HH10-11 embryos were transplanted into the posterior spinal cord of stage-matched wild type embryos. A sharpened tungsten needle or pulled glass capillary was used to peel back the vitelline membrane of donor and host embryos and to dissect out a small patch of dorsal cells from either the hindbrain or spinal cord. Tissue patches were transferred using a Gilson pipette and Ringers solution to the host embryos. A small incision was made into the posterior host embryo dorsal neural tube and tissue patches were pushed into this incision. Embryos were cultured *in ovo* overnight. Spinal cord to hindbrain grafts were attempted similarly.

#### Ferrofluid droplet experiments

Ferrofluid oil droplets (First4Magnets, EFH1 ferrofluid) were injected into the forebrain region of HH10-12 stage embryos using a Nanojet injector set to inject a volume of either 1, 2, or 4nl. Droplets were injected either at equal volumes (2nl) or at a ratio to account for the difference in initial lumen size (4nl in the larger hindbrain lumen, 1nl in the smaller spinal cord lumen). Droplets were positioned in the lumen of either the hindbrain or spinal cord using a magnet and custom-built embryo holder with embryos cultured *ex ovo*. Embryos were fixed approximately 1.5 hours post droplet injection prior to immunostaining and confocal imaging or imaged live, the droplet stayed positioned within the lumen throughout this process. Dorsal roof tissue thickness was measured at the point of maximum roof deformation in the hindbrain and spinal cord of injected embryos at the droplet location. Uninjected embryos at equivalent stages were measured at the same anterior-posterior level as controls. As a calibration control, hydrogel tubes (1% low-melt agarose) made with a microcapillary inside a microfluidics tube (Bio-Rad, Tygon tubing) were imaged following injection of a ferrofluid droplet inside.

#### Atomic force microscopy

Atomic force microscopy (AFM) indentation measurements were performed using the setup described in^56^. Briefly, HH10-12 stage chicken embryos were cultured on agarose plates and the vitelline membrane was removed to expose the embryo’s dorsal hindbrain and spinal cord. 89.3 μm diameter polystyrene beads were glued to Arrow-TL1 cantilevers (nominal spring constant = 0.01 N/m; NanoWorld, Neuchâtel, Switzerland) as probes. During measurements, the cantilever was approached with 10 μm/s until an indentation force of either 50nN or 75nN was reached. This force was maintained for 3 seconds, thus enabling the acquisition of the deformation over time (‘creep’).

In order to extract fluidity and elastic modulus values from the force-distance curves, ^57^ introduced the following piecewise model for the force ramp:

$$F(t)=F_{C}\{\begin{array}{cc} 0\;; & t<t_{c}\\ {\left(\frac{t-t_{C}}{\text{Δ}t_{A}}\right)}^{\alpha }; & t_{c}\leq t<t_{c}+\text{Δ}t_{A}\\ 1; & t\geq t_{c}+\text{Δ}t_{A} \end{array}$$

where *F_C_* is the magnitude of the applied force during the creep measurement reached in time *Δt_A_* and applied for time *t_C_*. α describes the shape of the force ramp (1 < *α* < 2), where α = 1 corresponds to a linear force ramp and α closer to 2 to a polynomial force ramp. Fitting this equation yields *F_C_, t_C_, Δt_A_*, and *α*. These values then derive the following equation for the indentation *δ*, here modified by substituting *E_0_*/(1- *v*^2^) with the reduced instantaneous elastic stiffness, *k_0_*

δ(t)=[341k0RFCα(t−tC)ΔtAαt0βα+βB(ΔtAt−tC;α,β+1)]23

where *R* is the radius of the bead, *B()* is the incomplete beta function and the exponent, and β is the fluidity, which can have values ranging from 0 (for an elastic solid) to 1 (for a viscous fluid). Mean values for *k_0_* and β were determined from measurements taken across the hindbrain and along the spinal cord. Values obtained for *k_0_* were close to values obtained for the reduced apparent elastic modulus *K* = *E_0_*/(1- *v*^2^) obtained by fitting the Hertz model 43Kδ32R to the instantaneous indentation data (Figure. S2G).

#### MMP inhibitor treatment

After removal of the vitelline membrane covering the brain, 20μl of 1000μM broad-spectrum MMP inhibitor (GM6001) or DMSO diluted in Ringers solution, was added to the dorsal surface of HH10-11 stage embryos *in ovo*. Embryos were incubated overnight.

#### Immunostaining

After fixing embryos overnight in 500μl 4% PFA at 4°C, they were washed 3X for ~20 minutes per wash in 0.5% Triton-X- 100 (Sigma-Aldrich, X100) in PBS (PBST). Embryos were incubated in blocking solution consisting of 4% Donkey serum in PBST for 1 hour at room temperature. Primary antibodies were diluted in blocking solution and incubated with embryos for 2 days at 4°C. After washing embryos in PBST ~4 times over the course of 2 hours, secondary antibodies, Hoecst and Phalloidin were diluted in blocking solution and incubated with embryos for another 2 days at 4°C. Finally, embryos were washed in PBST prior to imaging.

The following antibodies and dyes where used: Laminin (DSHB, 3H11) 1:100, Snail2 (Cell Signaling Technology, 9585S) 1:200, pMLC2 (Cell Signaling Technology, 3671S) 1:100, Hoechst 1:1000, Phalloidin 647 (Thermo Scientific, A22287) 1:500, Donkey Anti-Mouse 488n IgG (Abcam, ab150105), Donkey Anti-Rabbit 594 (Abcam, ab150076).

#### Confocal imaging

Embryo cross sections and wholemounts were mounted on glass-bottom confocal imaging dishes in VECTASHIELD (Vector Labs, H-1000-10) mounting medium. Z-stacks were acquired at either 0.3 or 0.5μm z-steps using either an SP8 (Leica) or a SoRa Spinning Disk (Nikon) using either a 10X, 20X or 40X objective.

### Quantification and statistical analysis

All data analysis was performed in Python (www.python.org), with data stored in Excel. Boxplots were created using the seaborn library and show the median, upper and lower quartiles and whiskers extending to 1.5 times the interquartile range. Scatterplots were plotted using matplotlib. Statistical analysis was performed using the scipy.stats Python library. Paired t-tests were used to calculate p-values for the AFM data. Independent t-tests were used to calculate all other p-values. All tests were two-sided and data checked for normality using the Shapiro-Wilk test. In all cases P<0.05 was taken as the threshold for significance. Measurements were acquired from distinct samples in all cases unless otherwise stated and n numbers are given in the figure legends. Details of measurements of reported features are listed below:

#### Tissue shape analysis

The thickness profile of neural tube cross-sections was acquired by manually tracing and measuring the radial tissue thickness along the lumen circumference starting from the approximate mid-point of the dorsal hindbrain roof. For wildtype characterisation, the distance along the lumen circumference was normalised for all samples so that tissue thickness at distance ‘0’ fell at the dorsal mid-point and tissue thickness at distance ‘1’ at the ventral midpoint of the neural tube cross section. A 4^th^ degree polynomial was fit to the thickness profiles to get an ‘average’ thickness profile for the hindbrain and spinal cord at HH11 and HH16. Images acquired by confocal microscopy were visualised using either ImageJ^58^ or napari^59^. Cross sections were either visualised directly, or by reordering the axes of image data acquired from wholemount samples in napari. For intubation experiment analysis the mean thickness of the first 100μm of dorsal roof tissue from the dorsal midpoint was compared. For BDX experiment analysis the mean thickness of the first 300μm of dorsal roof tissue from the dorsal midpoint was compared. Equivalent positions along the hindbrain were compared for assessing hindbrain roof thickness in MMP treated and control embryos and hindbrain roof length was obtained by measuring the length of the single-cell thick roof.

#### Actin and myosin intensity along apical surface

Curves were drawn along the apical surface of neural tube cross sections starting from the dorsal midpoint and moving ventrally. The actin or myosin intensity along the curve was measured using the ImageJ PlotProfile function. Normalised average intensities along the apical surface were obtained for each embryo and binned into two or three groups starting from the dorsal-most position and moving more ventrally. The mean intensity for each embryo within each bin was plotted.

#### Somite removal analysis

Z planes were selected in the region where somite removal had been performed and in a corresponding location in controls. Roof thickness was measured at the dorsal midpoint for z planes from each embryo.

## Supplementary Material

Movie S1

Movie S2

Supplementary Material

## Data Availability

Data: The published article includes most datasets generated during this study. Additional replicas and source datasets are available upon request. This study does not include data deposited in public repositories. Code: Custom codes used for data plotting are available upon request. All other requests: Any additional information required to reanalyze the data reported will be shared by the lead contact upon request.
