# Supplementary material for: Differential tissue deformability underlies fluid pressure driven shape divergence of the avian embryonic brain and spinal cord
Source: Dev Cell. Author manuscript; Available in PMC 2025 Nov 4. (PMC7618324; doi:10.1016/j.devcel.2025.04.010)
Supplement: Supplementary Material [file EMS209662-supplement-Supplementary_Material.pdf]

Supplemental Information

Document S1. Figures S1-S4 and Data S1

Movie S1. Droplet rounding in hindbrain, related to Figure 2 and S2

Droplet rounding in an HH11 stage wildtype chicken embryo hindbrain. Imaging started 6 minutes after injections were performed.

Movie S2. Droplet rounding in spinal cord, related to Figure 2 and S2

Droplet rounding in an HH11 stage wildtype chicken embryo spinal cord. Imaging started 6 minutes after injections were performed.

Document S1: Figures S1-S4

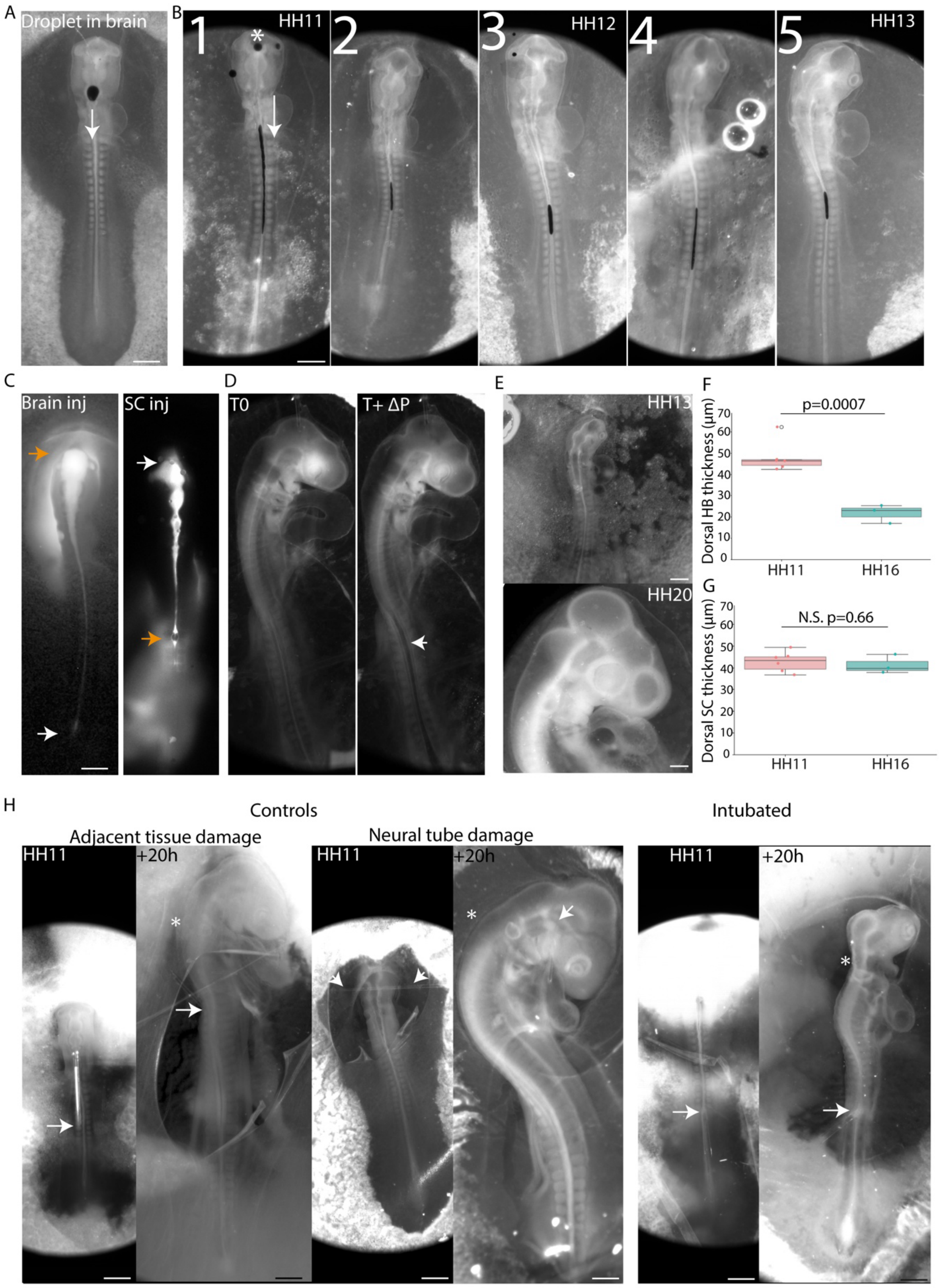

Figure S1. Neural tube characterisation and intubation experiments, related to Figure 1

(A) Image illustrating that a ferrofluid droplet can be pulled from the hindbrain into the spinal cord lumen using a magnetic field. (B) Images of embryos in stages leading up to brain expansion with droplets pulled from the brain into the spinal cord region. The droplets narrow as the entre the spinal cord lumen but are able to pass from the brain to spinal cord in each case. (C) Fluorescent dextran was injected into HH11 stage embryos at sites indicated by the orange arrows. Dye was able to flow both posteriorly into the spinal cord and anteriorly into the brain, with the extent of dye spreading indicated by the white arrows. (D) The spinal cord lumen responds immediately to an ~1500Pa increase in lumen pressure at the site of the midbrain (white arrow indicates spinal cord lumen opening on pressure increase) (E) Brightfield images of the anterior of embryos used in pressure measurements. (F) Mean thickness of the dorsal hindbrain in HH11 (n=6) and HH16 (n=3) stage embryos (p=0.0007, t-test). (G) Mean thickness of the dorsal spinal cord in HH11 (n=6) and HH16 (n=3) stage embryos (p=0.66, t-test). (H) Brightfield images of control and intubated embryos just after intubation and 20 hours later. White arrows mark site of intubation. White asterisks mark the hindbrain. All scale bars are 500μm.

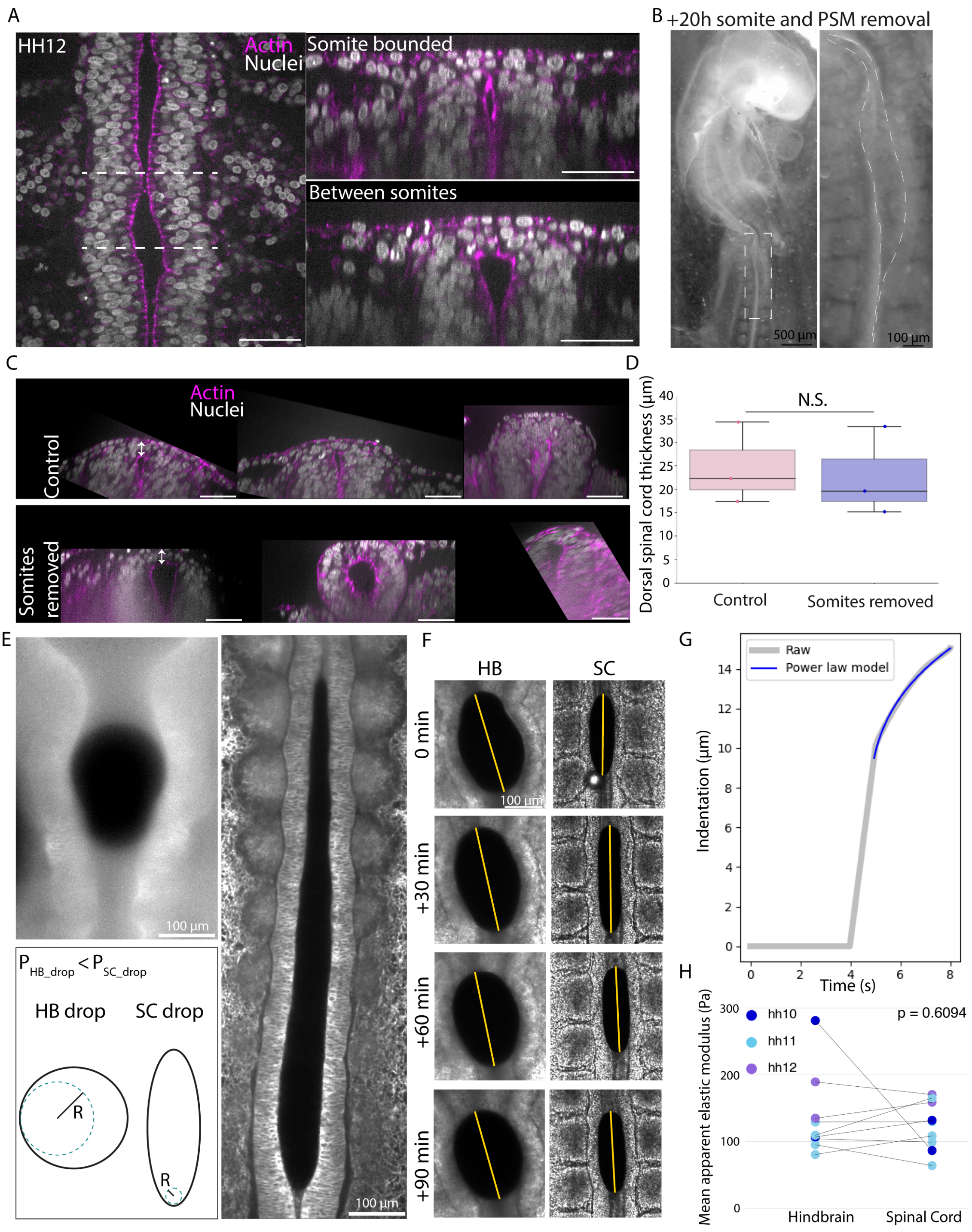

Figure S2. Geometry and tissue mechanics experiments, related to Figure 2

(A) Confocal images of dorsal and cross-sectional views of the spinal cord region in an HH12 stage embryo. Dashed lines indicate level of cross-sectional views. (B) A widefield image of an embryo ~20 hours after posterior somite and PSM removal with zoomed-in view of the widened spinal cord

region. (C) Confocal images of cross-sectional views of the spinal cord in embryos 20 hours after control cuts or somite removal. (D) Dorsal spinal cord thickness in control (n=3) and somite removed (n=3) embryos, measured at the dorsal midpoint ($p>0.05$, t-test). (E) Close up views of ferrofluid droplets in the hindbrain and spinal cord and a schematic illustrating the higher curvature at the droplet-lumen interface and thus higher pressure exerted by droplets in the spinal cord region compared to the hindbrain region. (F) Ferrofluid droplet rounding dynamics in the hindbrain and spinal cord. (G) Fitting of AFM indentation data to extract fluidity ‘β’. (H) Mean elastic modulus in the hindbrain and spinal cord of HH10-12 stage embryos (n=9, p=0.6094, t-test).

White scale bars are 50μm unless otherwise stated.

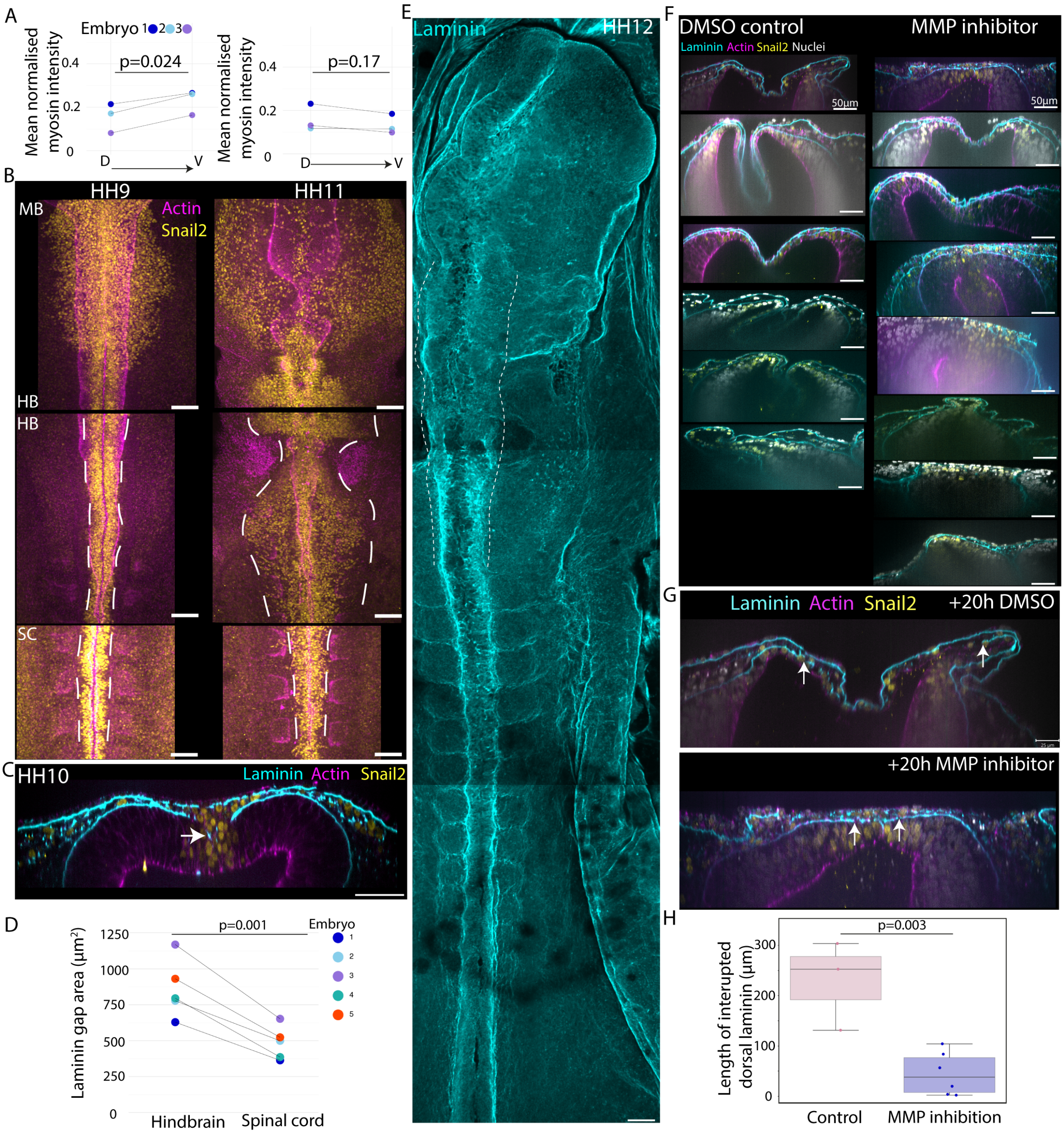

Figure S3. Neural crest and ECM experiments, related to Figure 3

(A) Quantification of apical myosin signal intensity along the lumen circumference in the hindbrain (left plot) and spinal cord (right plot) of HH11 and HH12 stage embryos (p=0.024 and p=0.17, t-tests). (B) Confocal images showing Snail2+ cells at the dorsal surface of HH9 and HH11 stage brain and spinal cord regions. Dashed lines depict the extent of Snail2+ cell spreading away from the dorsal midline. (C) Representative confocal image of ECM remodelling observed in an HH10 hindbrain cross section. (D) Total area of gaps in ECM on the dorsal surface of the hindbrain and spinal cord of

pre-brain expansion embryos (n=5) (p=0.001, t-test). (E) Confocal images stitched together to show a 3D rendering of the laminin matrix in a HH12 stage embryo. Dashed lines depict the boundaries of the hindbrain region. (F) Confocal images of DMSO and MMP inhibitor treated embryo hindbrains, ~20 hours post treatment. Actin not stained for in bottom 3 images in control and inhibitor panels. (G) Control and inhibitor treated hindbrains with arrows indicating interruption in the dorsal laminin surface. (H) Quantification of the length of the interrupted region of dorsal laminin in control (n=3) and inhibited (n=6) embryos (p=0.003, t-test). White scale bars are 100μm unless otherwise stated.

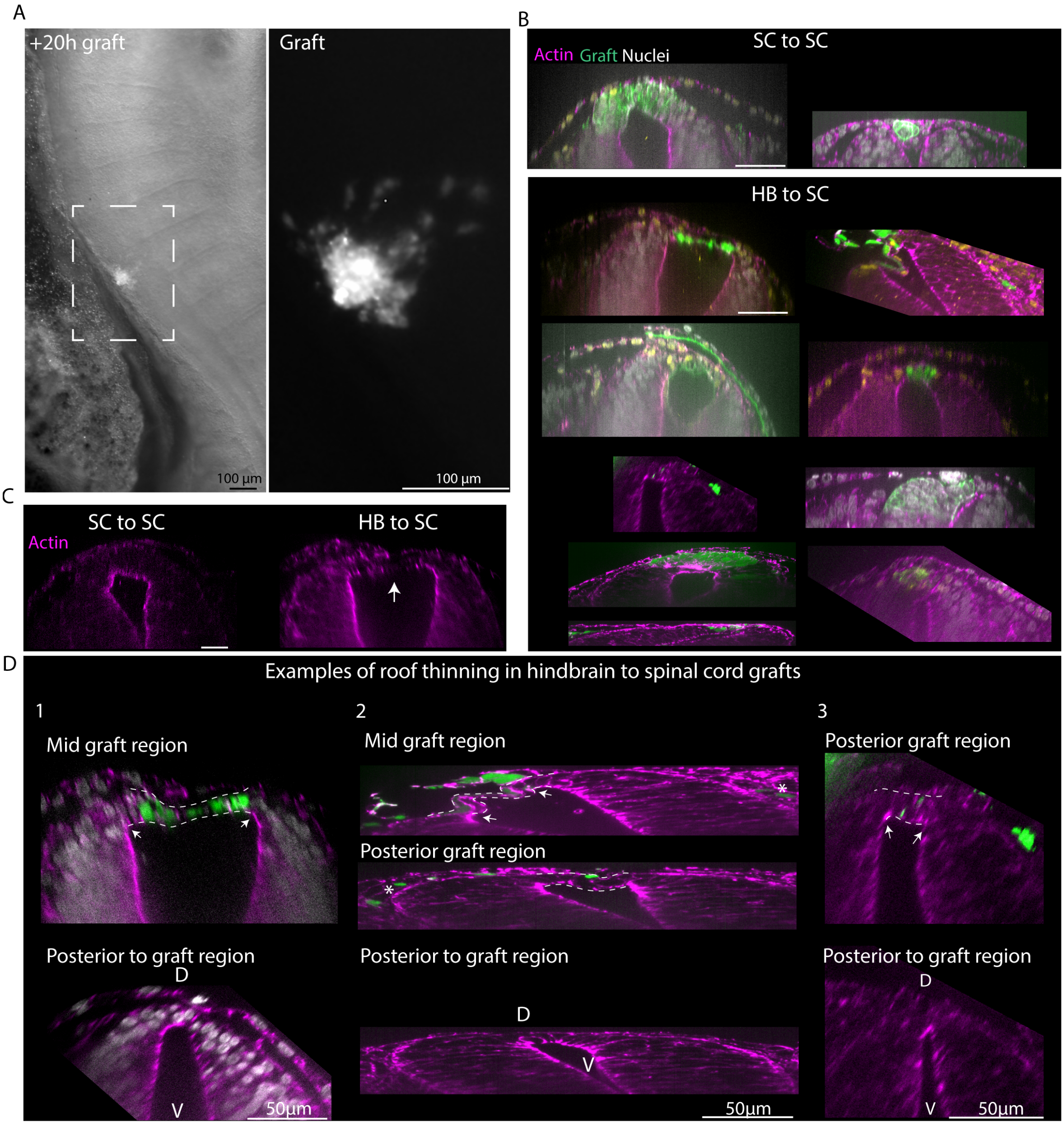

Figure S4. Graft experiments, related to Figure 4

(A) Widefield images of an embryo ~20 hours after introduction of a GFP+ graft into the spinal cord and a zoomed-in view of the graft region. (B) Confocal images of cross-sectional views of the spinal cord at spinal cord-spinal cord and hindbrain-spinal cord graft sites. (C) Confocal images showing actin organisation at the apical lumen surface in a spinal cord-spinal cord graft region and a hindbrain-spinal cord graft region. White arrow indicates loss of apical actin localisation. (D) Examples of dorsal tissue thinning in the spinal cord region following integration of grafted dorsal hindbrain cells. White scale bars are all 50µm unless otherwise stated.

Data S1: Tube deformation model under droplet pressure, related to Figure 2

In this Supplementary theory note, we propose a rheological model of neural tube expansion, including two possible deformation mechanisms: elastic stretching and viscous flowing, taking into account the geometrical differences between the hindbrain and the spinal cord. We also provide an approach to quantify the rheological parameters of tubular tissues by tracking the shape evolution of injected ferrofluid droplets. Based on the quantification of rheological parameters, we show that luminal fluid pressure can play a significant role in driving viscous long-term hindbrain expansion (Figure 1) and identify a ~7-fold difference in long-term tissue viscosity between the hindbrain and the spinal cord. Finally, a more detailed model considering the dorsal-ventral inhomogeneity in tissue viscosity can well reproduce the dorsal thinning and elongation observed in experiments, further supporting the rheological mechanism we proposed.

1. Rheological model of neural tube

The neural tube epithelium is treated as a viscoelastic Maxwell medium, which acts as an elastic solid on short time-scales and a viscous fluid on long time-scales[60–63]. In the "spring-dashpot" representation (Figure ST1), the Maxwell medium behaves as a Newtonian fluid (viscous dashpot with viscosity η) in series with a Hookean solid (elastic spring with elastic modulus E) so that the total deformation is the sum of the elastic and viscous contributions.

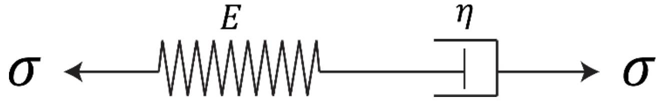

Figure ST1. Schematic representation of the Maxwell rheological model.

Driven by luminal fluid pressure or other forces (such as the mechanical interaction with the injected ferrofluid droplet in the lumen, see Figure 2D for instance, the tissue deformation includes two parts: elastic deformation and viscous flowing. Several considerations argue in favour of considering a long-term viscous rheology. Firstly, the neural tube (including both the hindbrain and spinal cord) shows only moderate morphological changes on a short time-scale (seconds to minutes), with the expansion effects of ferrofluid droplets occurring on long time scales of hours (Figure S2F, see also section 2 below). Secondly, a number of experimental and theoretical works have shown that epithelial tissues are well-described by long-term viscosity, due to multiple active events resulting in local

remodelling (e.g. divisions and intercalation)[60,61,64,65]. Thirdly, the luminal fluid pressure we measured is much lower than the expansion pressure engendered by the injected oil droplet, as well as orders of magnitude below epithelial stiffness, so that luminal fluid pressure is unlikely to lead to large elastic stretches during normal morphogenesis. In the modelling, we thus consider small elastic deformation characterized by elastic strain ϵ_α, with the subscript $\alpha = z, \theta, r$ respectively corresponding to the direction along the A-P axis, the azimuthal direction, and the radial direction.

Tissue deformation during neural tube morphogenesis can be characterized by comparing the current shape and the initial shape before the accumulation of luminal fluid (around Hamburg-Hamilton (HH) stage 11). The stretch ratio λ_α ($\alpha = z, \theta, r$), denoted as the ratio of the current length to the initial length of a tissue element along direction α, can evaluate the three-dimensional local deformation of the tissue. The stretch ratio λ_α of a viscoelastic tissue is contributed by both the elastic and viscous deformation. Consider the stretch ratio after elastic deformation is $1 + \epsilon_\alpha$, and denote the stretch ratio with only viscous flowing as q_α. Then the total stretch ratio λ_α is the product of these two parts:

$$\lambda_z = (1 + \epsilon_z)q_z, \qquad \lambda_\theta = (1 + \epsilon_\theta)q_\theta, \qquad \lambda_r = (1 + \epsilon_r)q_r. \tag{1}$$

The small elastic deformation of the tissue follows the linear elastic strain-stress relation, i.e. Hooke's law (Landau et al., 1986):

$$\begin{aligned}
\epsilon_z &= \frac{1}{E}[\sigma_z - v(\sigma_\theta + \sigma_r)], \\
\epsilon_\theta &= \frac{1}{E}[\sigma_\theta - v(\sigma_z + \sigma_r)], \\
\epsilon_r &= \frac{1}{E}[\sigma_r - v(\sigma_z + \sigma_\theta)],
\end{aligned} \tag{2}$$

with E the Young's modulus of the tissue, v the Poisson's ratio, and σ_α the elastic stress in direction α. Given that volume changes in cells involve forces much larger than those at play for morphogenetic shape changes[66], soft tissues are usually modelled as incompressible elastic media, which corresponds to $v = 1/2$.

As for Newtonian fluids, the rate of viscous deformation is proportional to shear stress. This relation can be extended to three-dimensional and large viscous deformation (see[67] for the theoretical framework of large viscoelastic deformation):

$$\frac{\dot{q_z}}{q_z} = \frac{1}{6\eta}(2\sigma_z - \sigma_\theta - \sigma_r),$$
$$\frac{\dot{q_\theta}}{q_\theta} = \frac{1}{6\eta}(2\sigma_\theta - \sigma_z - \sigma_r),$$
$$\frac{\dot{q_r}}{q_r} = \frac{1}{6\eta}(2\sigma_r - \sigma_z - \sigma_\theta), \tag{3}$$

with η the tissue viscosity. The stress σ_α in Eqs. (2) and (3) can be obtained by discussing the force balance condition in direction α.

2. Quantification of rheological parameters

Experimentally measuring long-term tissue mechanical properties has been an historic challenge in the field. Inspired by previous studies using embedded ferrofluid droplets to measure tissue mechanical properties (such as viscosity η and elastic modulus E) [28,62,68], we also inject ferrofluid droplets into the lumen of the neural tube and try to infer tissue rheology from droplet deformation. However, in previous studies, ferrofluid droplets were embedded within the bulk of tissues and thus sustained triaxial compressive stresses from the surrounding tissues, and corresponding quantitative relation between tissue rheology and droplet deformation cannot be applied to the neural tube, which has a tubular geometry which steers the droplet deformation in a different way. In this part, we provide theoretical foundations for the quantification of mechanics in tubular tissues.

2.1. Shape – pressure relationship of ferrofluid droplet

After being injected into the lumen of the neural tube, a ferrofluid droplet will deform into an ellipsoid due to the confinement from the tissue wall (Figure ST2). Its surface tension γ is balanced with the inner pressure p_d and an outer pressure, which is the luminal fluid pressure p_l in its polar region and tissue-droplet interfacial pressure p in its equator. The force balance in the fluid droplet also depends on the local curvatures:

$$2\gamma H_b = p_d - p_l \text{ (pole)}, \qquad 2\gamma H_a = p_d - p \text{ (equator)}, \tag{4}$$

with H_b and H_a respectively the mean curvatures in the pole and the equator (Figure ST2).

The above force balance links the tissue-droplet interfacial pressure p and the droplet inner pressure p_d with the droplet shape (surface curvature):

$$p_d = p_l + 2\gamma H_b\,, \qquad p = p_l + 2\gamma(H_b - H_a)\,. \tag{5}$$

For an ellipsoidal droplet with a and b respectively the lengths of its short and long axes, we have $H_b = 2b/a^2$ and $H_a = a/b^2 + 1/a$ [68]. Using Eq. (5), the pressures p and p_d can be inferred from the lengths of two major axes (i.e. a and b) of the ferrofluid droplet.

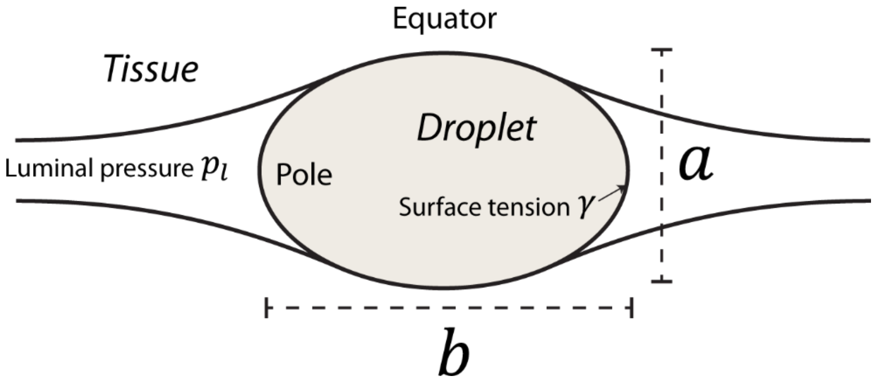

Figure ST2. Schematic geometry and mechanics of a ferrofluid droplet injected into the tissue lumen. The droplet with surface tension γ is deformed as an ellipsoid with short-axis length a and long-axis length b. Its equator (and nearby region) is in contact with the tubular tissue, while its pole is pressurized by luminal fluids (p_l).

2.2. Tissue viscosity of hindbrain inferred from droplet shape evolution

The hindbrain can be considered a thin-walled tissue, whose tissue thickness is smaller than tissue radius (approximately two or three times smaller than the tissue radius in HH11 and becomes approximately five times smaller than the radius in HH16, see Figure 1D for images of the tissue cross-sections). This allows us to approximate the hindbrain tissue as a viscoelastic membrane, where the stress in the radial direction σ_r is much smaller than the in-plane stresses (σ_θ and σ_z) and can be neglected. Thus, the deformation of a thin-wall tissue like the hindbrain is determined by the in-plane forces. Using the fact that the in-plane stresses in a thin wall tissue are homogeneously distributed along the thickness/radial direction, we can define in-plane tissue tensions $T_\theta = h\sigma_\theta$ and $T_z = h\sigma_z$ (h is the tissue thickness), which are the sum of in-plane stresses (σ_θ and σ_z) along the tissue thickness, and share the same unit (force per unit length) with the surface tension of ferrofluid droplet γ [69–72]. In this way, the interaction between the ferrofluid droplet and the viscoelastic hindbrain can be modelled as a two-layer membrane system (Figure ST3): a viscoelastic membrane with in-plane tension T_θ and T_z, and a liquid membrane with surface tension γ.

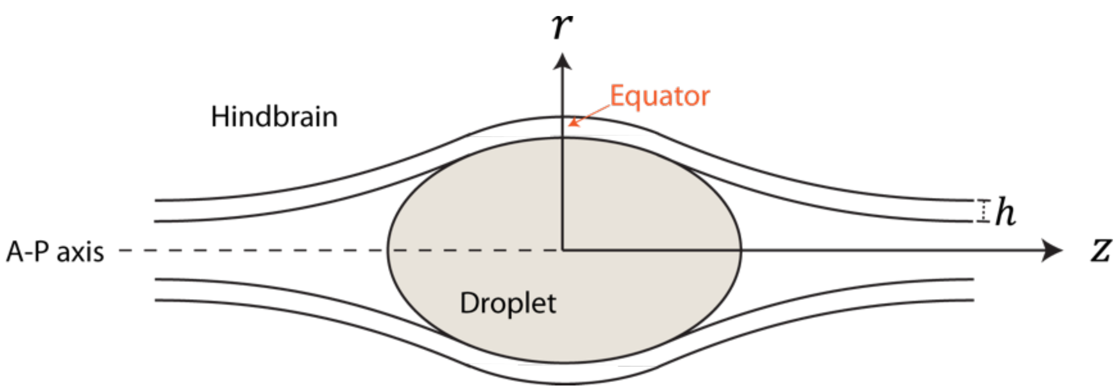

Figure ST3. Schematic of the hindbrain-droplet system.

The hindbrain-droplet system is cylindrically symmetric to the A-P axis. Note that in this geometry (Figure ST3), the axial stretching force T_z (σ_z) and corresponding deformation (e.g. λ_z and q_z) is in the meridional direction, not necessarily parallel to the A-P axis (i.e. z-axis). In such configuration, we have the azimuthal stretch ratio $\lambda_\theta = r/R$, with r and R respectively the current and initial radii of the hindbrain[73,74]. Importantly, the middle of the contact region (i.e. the droplet equator, see Figure ST3) of the hindbrain-droplet system satisfies a simple geometric relation: the current tissue radius is half length of the droplet's short axis (that is $r = a/2$), which directly links the tissue deformation and droplet shape.

Importantly, the shape evolution of the ferrofluid droplet depends on tissue viscosity. This is made clear from the fact that we observe droplet rounding to occur over time scales of hours, many orders of magnitude longer than what would be expected from rounding in water due to surface tension (Figure S2F). Thus, we can use the dynamics of droplet rounding as a proxy to estimate the long-term viscosity of the tissue, at the time scales of hours which are relevant for morphogenesis.

Considering the geometric relation $\dot{\lambda}_\theta/\lambda_\theta = \dot{a}/a$, and small elastic strains ϵ_α in Eq. (1), which leads to $\dot{\lambda}_\theta/\lambda_\theta \approx \dot{q_\theta}/q_\theta$, we can rewrite the second formula in the evolution equation (3) as

$$\frac{\dot{a}}{a} \approx \frac{1}{6\eta h}(2T_\theta - T_z). \tag{6}$$

Eq. (6) clearly shows the dependence of droplet shape on tissue viscosity η and in-plane tissue tensions T_θ and T_z, which can be obtained by discussing the force balance in the equator position (Figure ST3).

Similar to the force balance between surface tension and pressure in liquid droplets (e.g. Eq. (4)), the in-plane tissue tensions of the hindbrain are balanced with the interfacial

pressure p. Besides, in the meridional direction (i.e. z-direction in the droplet equator, see Figure ST3), the in-plane tissue tension T_z and droplet surface tension γ are balanced with the inner pressure of the droplet p_d. These lead to the following relations in the droplet equator:

$$T_\theta \kappa_{a\theta} + T_z \kappa_{az} = p,$$
$$2\pi r(T_z + \gamma) = \pi r^2 p_d \,, \tag{7}$$

with the principal curvature in the azimuthal direction $\kappa_{a\theta} = 2/a$, in the meridional direction $\kappa_{az} = 2a/b^2$, and tissue radius $r = a/2$ as aforementioned.

We introduce the aspect ratio of ferrofluid droplet $s = b/a$, so that the combination of Eqs. (5−7) gives the shape evolution equation of the ferrofluid droplet:

$$\frac{\dot{a}}{a} \approx \frac{1}{6\eta h}\left[\frac{1}{4}(3 - 2s^{-2})p_l a + (3s - 2s^{-1} - 1)\gamma\right]. \tag{8}$$

Considering the luminal fluid pressure $p_l \sim 15$ Pa (Figure 1C), lumen diameter in the hindbrain (or short-axis length of ferrofluid droplet) $a \sim 200$ μm, and the surface tension of ferrofluid $\gamma \sim 0.026\mathrm{N/m}$, one can find $p_l a/4 \ll \gamma$, and thus that the first term in the right side of shape evolution equation (8) can be neglected.

Note that the axis lengths a and b are not independent, but obey the geometric constraint of constant droplet volume $V_d = (4\pi/3)a^2 b$. With this constraint, the droplet shape (evolution) can be evaluated by a single shape parameter (a, b, or aspect ratio s), and these parameters are related to each other: for instance, we have

$$\frac{\dot{s}}{s} = -3\frac{\dot{a}}{a}. \tag{9}$$

By Eq. (9), we can replace the rate of shape evolution $\dot{a}/a$ with $\dot{s}/s$, and rewrite the shape evolution equation (8) as

$$\frac{\dot{s}}{s} \approx -\frac{\gamma}{2\eta h}(3s - 2s^{-1} - 1). \tag{10}$$

In Eq. (10), the tissue thickness also evolves with time, satisfying $\dot{h}/h = \dot{\lambda}_r/\lambda_r \approx \dot{q}_r/q_r$. Combined with the third formula in evolution equation (3), we can get the evolution law of tissue thickness. However, thickness evolution is quite slow compared with the shape change of the ferrofluid droplet, thus the tissue thickness h in Eq. (10) can be treated as a constant (which would bring quite small errors as shown in Figure ST5(a) but allows us to get analytic solutions). Then we can easily get the analytic solution of Eq. (10) as

$$\eta \approx -\frac{5\gamma}{2h}\frac{t_1 - t_0}{f_{\mathrm{hb}}(s_1) - f_{\mathrm{hb}}(s_0)}, \tag{11}$$

with $f_{\mathrm{hb}}(s) = \ln\left(\frac{s-1}{s+2/3}\right)$ and s_0 and s_1 respectively the aspect ratios of ferrofluid droplet in time points t_0 and t_1.

2.3. Tissue viscosity of spinal cord inferred from droplet shape evolution

Unlike the hindbrain, the spinal cord has a tissue thickness comparable with or even larger than its lumen radius. Besides, Figures 2B-C showed that the removal of surrounding tissues can enlarge the spinal cord, indicating the spinal cord is mechanically confined by surrounding tissues (such as somites). Thus, to discuss how the ferrofluid droplet mechanically interacts with the spinal cord, it is more realistic to consider its interaction with a broader mechanical surrounding (including the spinal cord and the surrounding tissue, see Figure ST4), compared to the thin-walled approximation employed to the hindbrain (see Subsection 2.2 for details). Let r_i and r_o respectively denote the inner and outer radius of the tissue system, we have $r_o \gg r_i = a/2$. In this scenario, the stress in A-P direction σ_z , which arises from droplet pressure p_d or luminal fluid pressure p_l is quite small, thus the tissue deformation is mainly driven by the azimuthal stress σ_θ and the radial stress σ_r.

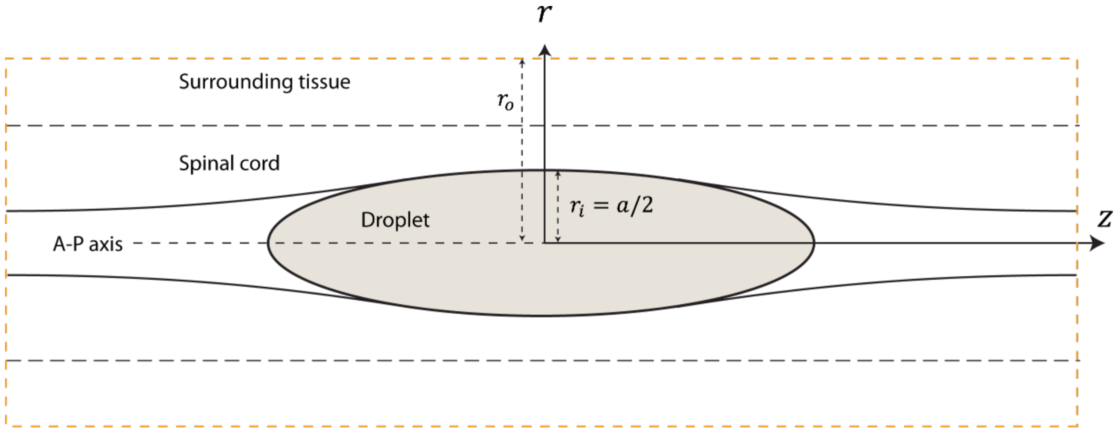

Figure ST4. Schematic of the spinal cord-droplet system.

Similar to the discussion in Subsection 2.2, we can get $\dot{\lambda}_\theta/\lambda_\theta = \dot{a}/a$ at $r = r_i = a/2$, and $\dot{\lambda}_\theta/\lambda_\theta \approx \dot{q}_\theta/q_\theta$ for small elastic strains. Using the evolution law of azimuthal viscous deformation in Eq. (3), we have

$$\frac{\dot{a}}{a} \approx \frac{1}{6\eta}(2\sigma_\theta - \sigma_r)|_{r=r_i}. \quad (12)$$

The tissue stresses σ_θ and σ_r are generated by the interfacial pressure p and radially distributed as [75]

$$\sigma_r = \frac{p r_i^2}{r_o^2 - r_i^2}\left(1 - \frac{r_o^2}{r^2}\right), \qquad \sigma_\theta = \frac{p r_i^2}{r_o^2 - r_i^2}\left(1 + \frac{r_o^2}{r^2}\right). \tag{13}$$

Considering $r_o \gg r_i$ leads to $(2\sigma_\theta - \sigma_r)|_{r=r_i} \approx 3p$ in Eq. (12). The interfacial pressure p can be inferred from the droplet shape by relation (5). Then, we can rewrite Eq. (12) as

$$\dot{a} \approx \frac{1}{\eta}\left[\frac{1}{2}p_l a + (2s - s^{-2} - 1)\gamma\right]. \tag{14}$$

The first term in the right of Eq. (14) can be neglected in data fitting, based on the scaling analysis that $p_l a$ is an order of magnitude smaller than droplet surface tension γ. Although the combination of Eqs. (9) and (14) can give close solutions of the shape evolution of ferrofluid droplet, they have to be solved numerically. Submitting Eq. (9) into Eq. (14), the shape evolution equation becomes:

$$\frac{\dot{s}}{s} \approx -3\frac{\gamma}{\eta a}(2s - s^{-2} - 1). \tag{15}$$

Eq. (9) indicates the aspect ratio s evolves faster than the short-axis length a, which allows us to approximate a as a constant. In this way, we can get the analytic formula for the data fitting of tissue viscosity of Eq. (15), which could be used to directly infer the tissue viscosity of the spinal cord from the shape evolution of droplet:

$$\eta \approx -\frac{6\gamma}{a}\frac{t_1 - t_0}{f_{\mathrm{sc}}(s_1) - f_{\mathrm{sc}}(s_0)}, \tag{16}$$

with $f_{\mathrm{sc}}(s) = \frac{3}{2\sqrt{7}}\arctan\left(\frac{1+4s}{\sqrt{7}}\right) + \frac{1}{4}\ln\left[\frac{(1-s)^2}{2s^2+s+1}\right]$. The complete solution of Eqs. (9) and (14) indicates the analytic formula (16) is accurate enough (Figure ST5(b)).

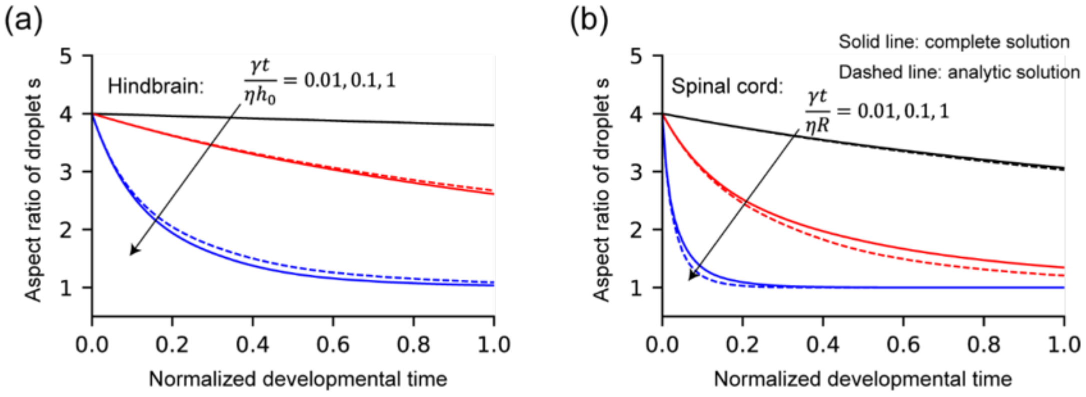

Figure ST5. Comparison between the complete and approximated solutions of the droplet shape evolution. (a) Time evolution of the aspect ratio of ferrofluid droplet in the hindbrain. Analytic solution in Eq. (11) treating tissue thickness h as a constant (i.e. $h = h_0$) is compared with the complete solution with thickness h also evolving. (b) Time evolution of

the aspect ratio of ferrofluid droplet in the spinal cord. Analytic solution in Eq. (16) considering lumen diameter (or short-axis length of droplet) a as a constant is compared with the complete solution.

3. Tissue deformation in normal development

Experimentally, we found that luminal fluids play an important role in the development of the neural tube, and the variation in luminal fluid pressure greatly affects the hindbrain deformation and morphogenesis (characterized by a thinning of the dorsal tissue and expansion of the lumen, see Figure 1). Here, we quantitatively discuss if the morphogenesis of the hindbrain could be a result of viscous or elastic deformation driven by luminal fluid pressure.

Importantly, we observe droplet rounding over hours after injection into the lumen of neural tube (Figure S2F), which is many orders of magnitude longer than what would be expected for droplets alone under surface tension (i.e. free from tissue confinement). This hints at a long-term viscous relaxation of tissue on the timescale of hours. Quantitatively, we tracked the tissue deformation rate $\dot{a}/a$ (with a the tissue diameter and $\dot{a}$ its rate) after injecting the ferrofluid droplet.

Driven by the droplet pressure, the hindbrain diameter increases by approximately 1.5X in 1-2 hours, suggesting the deformation rate $\dot{a}/a \sim \mathrm{h}^{-1}$. The viscous deformation rate of the hindbrain is proportional to in-plane tissue tension (Eq. (6)). The in-plane tension of the hindbrain expanded by the droplet is in the same order with the droplet surface tension γ, while in normal development, the hindbrain is expanded by the luminal fluid pressure p_l and the tissue tension is ~$p_l a$ based on Young-Laplace law (Fig. 2A) and Eq. (8). With the droplet surface tension $\gamma \sim 0.026\mathrm{N/m}$, luminal fluid pressure $p_l \sim 15\mathrm{Pa}$ and hindbrain diameter $a \sim 200\mu\mathrm{m}$, we found $p_l a$ is an order of magnitude smaller than γ, thus would speculate the viscous deformation rate in the normal development is an order of magnitude slower than the scenario with ferrofluid droplet, that is $\dot{a}/a \sim 0.1\mathrm{h}^{-1}$, which would leads to several folds of hindbrain expansion in around 20 hours (from HH11 to HH16), consistent with experimental observations (Figure 1).

Tissue viscosity η, the rheological parameter characterizing the tissue resistance to viscous flowing, can be inferred from the droplet shape evolution, as discussed in Subsection 2.2 and 2.3. Analytic formulas (11) and (16) provide a simple way to estimate the tissue viscosity η by comparing the aspect ratios in two different timepoints (usually with a time interval of one or two hours in our experimental setting). We estimate the tissue viscosity in

the hindbrain as 2.3$\pm$0.84 kPa $\cdot$ h (mean $\pm$SD), and that in the spinal cord as 17 $\pm$10 kPa $\cdot$ h. These values only provide an estimate and may diverge from the true viscosities of the neural tube owing to the droplet surface tension at the lumen interface being unknown. Experimental calibration of the model is challenging as manufacturing neural-tube-like small tubes using well-characterised materials is non-trival. We nonetheless created hydrogel tubes (1% low-melt agarose) using a microcapillary inside a microfluidics tube to be as small as possible (still orders of magnitude larger than the neural tube), followed by injecting the ferrofluid droplet inside. We observed very limited rounding of the droplets over a timescale of hours and inferred a viscosity of ~2.7kPa $\cdot$ h, which is consistent with this material behaving predominantly as a solid and are similar to measurements made for other hydrogels[76]. Moreover, these estimations are in line with our modelled results of the neural tube tissue (2-20kPa*h), showing consistency across tube/droplet sizes over a few orders of magnitude, lending further support to the validity of the model in assessing the relative differences of tissue regions. Importantly, our quantification shows that, the hindbrain is more fluidized and easier to deform than the spinal cord under mechanical loading. This is consistent with atomic force microscopy (AFM) measurement (Figure 2H) and molecular and cellular evidence supporting tissue fluidization in the hindbrain (Figure 3 and 4).

The minimal deformation of the hindbrain tissue observed immediately following droplet placement suggests a small degree of elastic deformation. In the normal development with the hindbrain expansion driven by luminal fluid pressure, the elastic deformation of the tissue would be even smaller, far less than enough to reproduce the several folds of hindbrain expansion observed in normal morphogenesis (Figure 1).

The viscous evolution laws (e.g. Eqs. (8) and (14)) in Section 2 include both the contributions from the luminal fluids and ferrofluid droplet, while in the normal development, the luminal fluid pressure p_l is the only driving force for tissue deformation. After removing the impact of the droplet (we could set $\gamma = 0$ and $s \to \infty$), the sole impact of the luminal pressure p_l on the viscous evolution or elastic displacement can be obtained:

- time evolution rate of tissue diameter $\dot{a}$ due to tissue viscous deformation:

$$\dot{a} \approx \frac{p_l a^2}{8\eta h} \text{ (hindbrain)}, \qquad \dot{a} \approx \frac{p_l a}{2\eta} \text{ (spinal cord)}. \tag{17}$$

4. Rheological inhomogeneity in the hindbrain

In the previous analysis and discussion, the tissue is considered to be axially varying (i.e. the geometric and mechanical properties may vary along A-P axis) but are circumferentially homogeneous. However, in the normal development, the dorsal and ventral regions of the hindbrain show distinct morphological evolution (Figure 1D). In the main text, this morphological difference along D-V axis is attributed to higher deformability of the dorsal tissue compared with its ventral partner (Figure 3 and 4). Tissue fluidization driven by neural crest ECM remodelling in the dorsal hindbrain would decrease the tissue viscosity and make the dorsal hindbrain easier to deform under mechanical load. To quantitatively test this rheological assumption, here we extend the model to consider the D-V inhomogeneity in tissue viscosity.

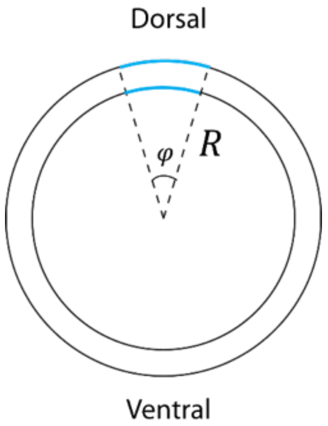

Figure ST6. Schematic of the cross-section of the hindbrain.

In the cross-section of the hindbrain (see Figure ST6 for schematic), consider the dorsal and ventral regions respectively with initial arc lengths $L_D = 2\pi\varphi R$ and $L_V = 2\pi(1-\varphi)R$ (φ is the initial length ratio of the dorsal to ventral region), and the same initial tissue thickness h_0, but different tissue viscosities, i.e. $\eta_D < \eta_V$. As a viscoelastic membrane with deformation dominated by viscous flowing (see Subsection 2.2 for details), the time evolution of the stretch ratios of the hindbrain tissue yields

$$\frac{\dot{\lambda}_{z\beta}}{\lambda_{z\beta}} \approx \frac{1}{6\eta_\beta h_\beta}(2T_{z\beta} - T_{\theta\beta}),$$
$$\frac{\dot{\lambda}_{\theta\beta}}{\lambda_{\theta\beta}} \approx \frac{1}{6\eta_\beta h_\beta}(2T_{\theta\beta} - T_{z\beta}),$$
$$\frac{\dot{\lambda}_{r\beta}}{\lambda_{r\beta}} \approx -\frac{1}{6\eta_\beta h_\beta}(T_{z\beta} + T_{\theta\beta}), \quad (18)$$

with $\beta = D, V$ respectively standing for the dorsal and ventral region of the hindbrain.

The azimuthal stretch $\lambda_{\theta\beta}$ is the ratio of current arc length l_β to its initial value L_β, that is $\lambda_{\theta\beta} = l_\beta/L_\beta$. The regional arc lengths are related to the current tissue radius r, and should satisfy the geometric constraint: $l_D + l_V = 2\pi r$. We can easily get $\dot{\lambda}_{\theta\beta}/\lambda_{\theta\beta} = \dot{l}_\beta/l_\beta$ in Eq. (18). Similarly, the radial stretch ratio $\lambda_{r\beta}$ evaluates the change of tissue thickness h_β and we have $\dot{\lambda}_{r\beta}/\lambda_{r\beta} = \dot{h}_\beta/h_\beta$. In the A-P axial direction, the deformations of the dorsal and ventral regions are synchronous: $\lambda_{zD} = \lambda_{zV}$ and $\dot{\lambda}_{zD} = \dot{\lambda}_{zV}$.

The in-plane tissue tensions are balanced with the luminal fluid pressure p_l. The dorsal and ventral hindbrains sustain the same azimuthal tissue tension: $T_{\theta D} = T_{\theta V} = T_\theta = p_l r$, and their total axial forces (along A-P axis) are balanced with $p_l \cdot \pi r^2$: $T_{zD} l_D + T_{zV} l_V = p_l \cdot \pi r^2$.

It is convenient to non-dimensionalize the parameters: we have tissue geometric parameters $\bar{r} = r/R$, $\bar{l}_\beta = l_\beta/R$, and $\bar{h}_\beta = h_\beta/h_0$, normalized tissues tensions $\bar{T}_\theta = T_\theta/(p_l R)$, $\bar{T}_{z\beta} = T_{z\beta}/(p_l R)$. The evolution of the hindbrain morphology depends on two parameters: the viscosity inhomogeneity $\Lambda = \eta_D/\eta_V$ and a dimensionless time $\tau = pRt_*/(6\eta_V h_0)$, with t_* the total developmental time. Considering luminal fluid pressure $p_l \approx 15\text{Pa}$, initial radius to thickness ratio $R/h_0 \approx 2$, the developmental time $t_* \approx 20\text{h}$ and ventral tissue viscosity $\eta_V {\sim} 10^3 \text{Pa} \cdot \text{h}$, we can estimate $\tau{\sim}0.1$. The final governing equation system includes three parts (with the upper bars for quantities dropped):

- time evolution laws for arc lengths and tissue thicknesses:

$$\begin{aligned} \dot{l}_D &= \frac{\tau l_D}{\Lambda h_D}(2T_\theta - T_{zD}), \qquad & \dot{l}_V &= \frac{\tau l_V}{h_V}(2T_\theta - T_{zV}), \\ \dot{h}_D &= -\frac{\tau}{\Lambda}(T_\theta + T_{zD}), \qquad & \dot{h}_V &= -\tau(T_\theta + T_{zV}). \end{aligned} \tag{19}$$

- force balance conditions:

$$T_\theta = r, \qquad T_{zD} l_D + T_{zV} l_V = \frac{r^2}{2}, \tag{20}$$

- geometric constraints:

$$l_D + l_V = r, \qquad 2T_{zD} - T_\theta = \frac{\Lambda h_D}{h_V}(2T_{zV} - T_\theta). \tag{21}$$

Interestingly, the rheological model considering a lower tissue viscosity in the dorsal hindbrain can well reproduce the morphological features observed in the morphogenesis of neural tube (Figure ST7(a)): the dorsal hindbrain becomes much thinner than the ventral hindbrain with development (Figure ST7(b)), meanwhile the arc length of the dorsal hindbrain elongates with time (Figure ST7(c)). This further validates that the rheological

model can well describe the morphogenesis of neural tube and tissue rheological behaviours respond to the morphological features.

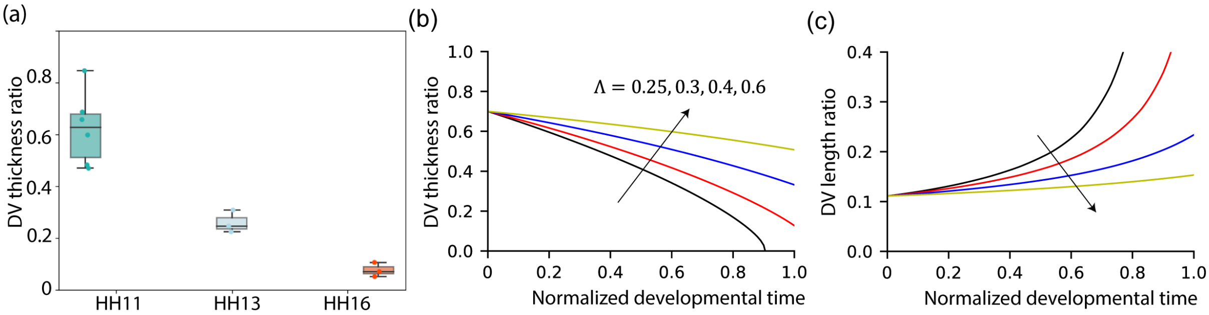

Figure ST7. The time evolution of dorsal to ventral thickness ratio and length ratio with development. This numerical example sets the dimensionless parameter $\tau = 0.1$, and varies the viscosity inhomogeneity Λ.

Supplemental References

1. Guevorkian, K., Colbert, M.-J., Durth, M., Dufour, S., and Brochard-Wyart, F. (2010). Aspiration of biological viscoelastic drops. Phys. Rev. Lett. *104*, 218101. https://doi.org/10.1103/PhysRevLett.104.218101.

2. Ranft, J., Basan, M., Elgeti, J., Joanny, J.-F., Prost, J., and Jülicher, F. (2010). Fluidization of tissues by cell division and apoptosis. Proceedings of the National Academy of Sciences *107*, 20863–20868. https://doi.org/10.1073/pnas.1011086107.

3. Serwane, F., Mongera, A., Rowghanian, P., Kealhofer, D.A., Lucio, A.A., Hockenbery, Z.M., and Campàs, O. (2017). In vivo quantification of spatially varying mechanical properties in developing tissues. Nat Meth *14*, 181–186. https://doi.org/10.1038/NMETH.4101.

4. Karkali, K., Tiwari, P., Singh, A., Tlili, S., Jorba, I., Navajas, D., Muñoz, J.J., Saunders, T.E., and Martin-Blanco, E. (2022). Condensation of the Drosophila nerve cord is oscillatory and depends on coordinated mechanical interactions. Dev Cell *57*, 867-882.e5. https://doi.org/10.1016/j.devcel.2022.03.007.

5. Petridou, N.I., Grigolon, S., Salbreux, G., Hannezo, E., and Heisenberg, C.-P. (2019). Fluidization-mediated tissue spreading by mitotic cell rounding and non-canonical Wnt signalling. Nat Cell Biol *21*, 169–178. https://doi.org/10.1038/s41556-018-0247-4.

6. Jain, A., Ulman, V., Mukherjee, A., Prakash, M., Cuenca, M.B., Pimpale, L.G., Münster, S., Haase, R., Panfilio, K.A., Jug, F., et al. (2020). Regionalized tissue fluidization is required for epithelial gap closure during insect gastrulation. Nat Commun *11*, 5604. https://doi.org/10.1038/s41467-020-19356-x.

7. Salbreux, G., Charras, G., and Paluch, E. (2012). Actin cortex mechanics and cellular morphogenesis. Trends in Cell Biology *22*, 536–545. https://doi.org/10.1016/j.tcb.2012.07.001.

8. Reese, S., and Govindjee, S. (1998). A theory of finite viscoelasticity and numerical aspects. International Journal of Solids and Structures *35*, 3455–3482. https://doi.org/10.1016/S0020-7683(97)00217-5.

9. Mongera, A., Rowghanian, P., Gustafson, H.J., Shelton, E., Kealhofer, D.A., Carn, E.K., Serwane, F., Lucio, A.A., Giammona, J., and Campàs, O. (2018). A fluid-to-solid jamming transition underlies vertebrate body axis elongation. Nature *561*, 401. https://doi.org/10.1038/s41586-018-0479-2.

10. Doubrovinski, K., Swan, M., Polyakov, O., and Wieschaus, E.F. (2017). Measurement of cortical elasticity in Drosophila melanogaster embryos using ferrofluids. Proceedings of the National Academy of Sciences *114*, 1051–1056. https://doi.org/10.1073/pnas.1616659114.

11. Skalak, R., Tozeren, A., Zarda, R.P., and Chien, S. (1973). Strain Energy Function of Red Blood Cell Membranes. Biophys J *13*, 245–264.

12. Srivastava, A., Tepole, A.B., and Hui, C.-Y. (2016). Skin stretching by a balloon tissue expander: Interplay between contact mechanics and skin growth. Extreme Mechanics Letters *9*, 175–187. https://doi.org/10.1016/j.eml.2016.06.008.

13. Goriely, A. (2017). The Mathematics and Mechanics of Biological Growth (Springer) https://doi.org/10.1007/978-0-387-87710-5.

14. Gómez-González, M., Latorre, E., Arroyo, M., and Trepat, X. (2020). Measuring mechanical stress in living tissues. Nat Rev Phys *2*, 300–317. https://doi.org/10.1038/s42254-020-0184-6.

15. Knoche, S., and Kierfeld, J. (2011). Buckling of spherical capsules. Phys. Rev. E *84*, 046608. https://doi.org/10.1103/PhysRevE.84.046608.

16. Haas, P.A., and Goldstein, R.E. (2015). Elasticity and glocality: initiation of embryonic inversion in Volvox. Journal of The Royal Society Interface *12*, 20150671. https://doi.org/10.1098/rsif.2015.0671.

17. Landau, L. D, Lifshitz, E. M., Kosevich, A. M., and Pitaevskii, L. P Theory of Elasticity - 3rd Edition (Elsevier).

18. Galli, M., Comley, K.S.C., Shean, T.A.V., and Oyen, M.L. (2009). Viscoelastic and poroelastic mechanical characterization of hydrated gels. Journal of Materials Research *24*, 973–979. https://doi.org/10.1557/jmr.2009.0129.
